# Supplementary material for: Benchmark Study on Phosphorescence Energies of Anthraquinone Compounds: Comparison between TDDFT and UDFT
Source: Molecules. 2023 Apr 5;28(7):3257. doi: 10.3390/molecules28073257 (PMC10096341; doi:10.3390/molecules28073257)
Supplement: Supplementary file 1 [file molecules-28-03257-s001.zip › molecules-2289708-supplementary.pdf]

Benchmark Study on Phosphorescence Energies with TDDFT and UDFT against Anthraquinone Compounds

Yujie Guo, Lingyu Zhang, Zexing Qu*

Institute of Theoretical Chemistry, College of Chemistry, Jilin University, Changchun, 130023, China

* Corresponding author e-mail:

zxqu@jlu.edu.cn

Contents:

Cartesian Coordinates

Table S1. Cartesian coordinates (Å) of optimized geometries.

HA Singlet

C	-0.86001100	-3.85868000	0.00000000
C	0.28996900	-3.09101700	0.00000000
C	0.20016100	-1.67645400	0.00000000
C	-1.07841500	-1.05679600	0.00000000
C	-2.22799100	-1.86321200	0.00000000
C	-2.12745300	-3.24591500	0.00000000
C	1.37196600	-0.84561200	0.00000000
C	-1.21957500	0.40438900	0.00000000
C	0.00000000	1.19620900	0.00000000
C	1.29115100	0.58812400	0.00000000
C	2.45597800	1.38425400	0.00000000
H	3.43495000	0.91806100	0.00000000
C	2.33675700	2.76884900	0.00000000
C	1.08788000	3.39031700	0.00000000
C	-0.08263300	2.61877500	0.00000000
H	-0.78138900	-4.94213500	0.00000000
H	1.26410300	-3.56903400	0.00000000
H	-3.19052500	-1.36251900	0.00000000
H	-3.02517000	-3.85703600	0.00000000
H	3.23462600	3.38062200	0.00000000
H	0.99046900	4.47072800	0.00000000
O	-2.35970700	0.93826500	0.00000000
O	2.54743200	-1.41665000	0.00000000
O	-1.26155700	3.24727600	0.00000000
H	-1.96312000	2.53080700	0.00000000

HA Triplet

C	-0.86097600	-3.85842300	0.00000000
C	0.28864500	-3.09084400	0.00000000
C	0.19683700	-1.67681900	0.00000000
C	-1.08021800	-1.05651300	0.00000000
C	-2.23058700	-1.86322300	0.00000000
C	-2.12934400	-3.24575400	0.00000000
C	1.37357700	-0.84749600	0.00000000
C	-1.22002100	0.40498000	0.00000000
C	0.00000000	1.19691300	0.00000000
C	1.29014600	0.58956000	0.00000000
C	2.45647200	1.38277000	0.00000000
H	3.43189400	0.90747200	0.00000000
C	2.33833500	2.76719500	0.00000000
C	1.08976100	3.39052300	0.00000000
C	-0.08195000	2.61973600	0.00000000
H	-0.78264200	-4.94198200	0.00000000
H	1.26711400	-3.56152400	0.00000000
H	-3.19320200	-1.36238500	0.00000000
H	-3.02681800	-3.85719900	0.00000000
H	3.23655000	3.37866100	0.00000000
H	0.99348700	4.47100700	0.00000000
O	-2.35970500	0.93975700	0.00000000
O	2.55171000	-1.41887800	0.00000000
O	-1.26041000	3.24869700	0.00000000
H	-1.96321600	2.53370800	0.00000000

AAT Singlet

C	-3.94100800	0.38496800	0.00007900
C	-2.84435400	1.22728600	0.00013900
C	-1.53470500	0.70315400	0.00001500
C	-1.35396400	-0.70842900	-0.00017800
C	-2.48794400	-1.55071400	-0.00021400
C	-3.76208700	-1.01477800	-0.00010500
C	-0.39675800	1.61982100	0.00011600
C	-0.02533000	-1.30513500	-0.00025300
C	1.11002800	-0.37751900	-0.00010000
C	0.92677800	1.01545500	0.00009600
C	2.07554500	1.89619000	0.00008600
H	1.86465200	2.95995900	0.00039500
C	3.37017400	1.40649000	-0.00033600
C	3.58674200	0.02587500	-0.00055800
C	2.47313300	-0.86897400	0.00005900
H	-4.94526400	0.80091400	0.00019800
H	-2.95497500	2.30670900	0.00029200
H	-2.32550000	-2.62335000	-0.00035200
H	-4.62817300	-1.67130400	-0.00018500
H	4.21637000	2.08632200	-0.00067600
H	4.59089300	-0.38952100	-0.00080100

O	0.13935900	-2.56390500	-0.00024500
O	-0.52444900	2.87259100	0.00022200
H	3.60343500	-2.58223400	0.00031200
N	2.67050400	-2.18907000	0.00103800
H	1.78824600	-2.75564600	0.00068000

AAT Triplet

C	-3.94032500	0.37757700	-0.00002700
C	-2.84490200	1.22312400	0.00013900
C	-1.53594500	0.70148700	0.00010000
C	-1.35050300	-0.70785300	-0.00017600
C	-2.48041800	-1.55338500	-0.00035700
C	-3.75683700	-1.02012900	-0.00026700
C	-0.39685200	1.62576300	0.00021300
C	-0.01615300	-1.30206800	-0.00018200
C	1.10893800	-0.37714700	-0.00052900
C	0.92399300	1.02276000	0.00007600
C	2.06762100	1.89445500	-0.00010700
H	1.85915400	2.95951200	0.00004500
C	3.36989700	1.40551900	-0.00050000
C	3.59007300	0.02857700	-0.00065300
C	2.47749200	-0.86785100	0.00005700
H	-4.94577200	0.79064700	0.00004000
H	-2.95713400	2.30239300	0.00026400
H	-2.31477500	-2.62550400	-0.00056300
H	-4.62088100	-1.67950300	-0.00042900
H	4.21238100	2.08975400	-0.00108600
H	4.59486400	-0.38510000	-0.00066100
O	0.14432000	-2.56401000	0.00002000
O	-0.53788700	2.87410200	0.00068900
H	3.59624500	-2.58618300	0.00065600
N	2.66528500	-2.18658800	0.00117600
H	1.77099200	-2.74562000	0.00112800

BP Singlet

C	-0.00013700	0.92858800	-0.00016400
O	0.00061000	2.26099500	-0.00069600
C	1.29973200	0.30823900	-0.01912300
C	1.50815500	-0.99094800	-0.55850300
C	2.43738600	1.03032900	0.44324600
C	2.77532800	-1.55242100	-0.57405600
H	0.66920200	-1.53559300	-0.97868200
C	3.70115700	0.45790600	0.40633900
H	2.29709800	2.02397300	0.85391400
C	3.88381900	-0.83728700	-0.09275600
H	2.91110800	-2.55095500	-0.98142400
H	4.55306600	1.02029400	0.77959700
H	4.87407000	-1.28265900	-0.11580700

C	-1.29982000	0.30826600	0.01899500
C	-1.50806500	-0.99115000	0.55786300
C	-2.43765500	1.03052700	-0.44259200
C	-2.77528100	-1.55258300	0.57372500
H	-0.66902300	-1.53604000	0.97754100
C	-3.70141200	0.45813000	-0.40544200
H	-2.29757000	2.02436800	-0.85289300
C	-3.88392800	-0.83728800	0.09317500
H	-2.91086500	-2.55127500	0.98077700
H	-4.55344500	1.02070700	-0.77814000
H	-4.87418900	-1.28262400	0.11643200

BP Triplet

C	0.00004300	0.92006600	-0.00003100
O	-0.00017500	2.24963500	-0.00027700
C	1.30687000	0.30017400	0.01699500
C	1.51906600	-0.99439800	0.55624700
C	2.43535600	1.02802000	-0.44776800
C	2.79108100	-1.54840500	0.57313700
H	0.68211200	-1.54525800	0.97231500
C	3.70471700	0.46542500	-0.40706900
H	2.28694600	2.01944600	-0.86257000
C	3.89431400	-0.82646000	0.09426100
H	2.93214900	-2.54698100	0.97867300
H	4.55328200	1.03352100	-0.77927000
H	4.88740500	-1.26561900	0.11896400
C	-1.30684100	0.30016900	-0.01701700
C	-1.51910000	-0.99431900	-0.55646100
C	-2.43526500	1.02793800	0.44801900
C	-2.79110700	-1.54832600	-0.57326700
H	-0.68218200	-1.54510100	-0.97270300
C	-3.70463800	0.46534800	0.40738300
H	-2.28679900	2.01928300	0.86298400
C	-3.89428800	-0.82644600	-0.09413600
H	-2.93223600	-2.54684400	-0.97892700
H	-4.55315400	1.03338000	0.77978900
H	-4.88737600	-1.26561300	-0.11878700

TX-BT Singlet

C	4.67363100	-0.41860900	-0.96332200
C	3.64066100	-1.21797000	-0.47502700
C	2.53152300	-0.62330100	0.13498900
C	2.44106700	0.77166400	0.26223200
C	3.47194200	1.55638100	-0.26324300
C	4.58637900	0.97109800	-0.86526600
C	1.25725200	1.37616100	0.98667000
C	-0.06609700	0.77279800	0.56485800
C	-0.18683500	-0.62360200	0.47311700

C	-1.41525100	-1.23463500	0.14138300
H	-1.47381200	-2.31364000	0.03605000
C	-2.50868100	-0.42945900	-0.10299900
C	-2.42834700	0.98851600	-0.00466500
C	-1.18744700	1.56667100	0.30644700
H	5.53569400	-0.88211000	-1.43499300
H	3.68660900	-2.29855300	-0.57651000
H	3.40505600	2.63904600	-0.18293100
H	5.38204800	1.59822900	-1.25750000
H	1.24065600	2.46194500	0.84751700
H	-1.10437500	2.64861600	0.38103100
S	1.22707700	-1.66685800	0.77604100
C	-3.68249100	1.63060500	-0.29101900
S	-4.14622100	-0.98913000	-0.64694000
C	-4.80878000	0.61987100	-0.20005500
H	-3.78080000	2.56931500	-0.82731100
H	-5.48471700	0.63693500	0.65691300
H	1.38880000	1.19889200	2.06752200

TX-BT Triplet

C	4.73740200	-0.42359800	-0.89988300
C	3.67704700	-1.22005400	-0.47190800
C	2.54545000	-0.62403000	0.09631700
C	2.45988700	0.76880500	0.25092900
C	3.52511100	1.54913800	-0.21015700
C	4.65843400	0.96494000	-0.77624600
C	1.26664300	1.38625900	0.94827700
C	-0.06332200	0.78354200	0.54341800
C	-0.17717400	-0.62276100	0.45480400
C	-1.47022300	-1.25992800	0.18671100
H	-1.53238500	-2.34269000	0.14710300
C	-2.54493700	-0.46354500	-0.03859500
C	-2.46571700	0.98616300	0.00015600
C	-1.16974300	1.57842500	0.31415200
H	5.61444900	-0.88680800	-1.34307300
H	3.71762100	-2.29922500	-0.59114000
H	3.46735100	2.63044800	-0.10857500
H	5.47618400	1.59195400	-1.12015600
H	1.25445400	2.46794200	0.78059800
H	-1.08921500	2.65963100	0.38352700
S	1.20786000	-1.68226900	0.62462300
C	-3.66568900	1.59465800	-0.26589100
S	-4.21310200	-0.98509700	-0.42787200
C	-4.74847000	0.67464800	-0.52743800
H	-3.81342700	2.66971300	-0.28128700
H	-5.79182700	0.90945200	-0.67734800
H	1.39246800	1.24146200	2.03446900

TX-DBT Singlet

C	5.94363100	-0.09077600	0.00040300
C	4.91117800	-1.02303900	0.00004400
C	3.57993300	-0.59645200	-0.00006300
C	3.23364300	0.79560200	0.00007400
C	4.31802600	1.71721400	0.00053700
C	5.62933000	1.28435600	0.00068900
C	1.88218300	1.27211000	-0.00028300
C	0.72181100	0.42036100	-0.00032200
C	0.80414700	-1.01913100	-0.00017700
C	-0.33347300	-1.82582600	0.00002600
H	-0.23576900	-2.90780300	0.00013800
C	-1.58649300	-1.22007600	0.00000500
H	6.97716000	-0.42164400	0.00051800
H	5.13419400	-2.08708200	-0.00017700
H	4.07344600	2.77238600	0.00073200
H	6.43204800	2.01713800	0.00105600
S	2.34964400	-1.85806100	-0.00055500
C	-3.10558000	0.61428700	-0.00012500
C	-3.99319400	-0.48533900	0.00020000
C	-5.37584000	-0.30687000	0.00043800
C	-5.87767100	0.99451800	0.00028500
C	-5.01245700	2.09856400	-0.00009800
C	-3.63387900	1.91415300	-0.00030100
H	-6.04855200	-1.15939300	0.00076700
H	-6.95268900	1.15126600	0.00049100
H	-5.42202200	3.10438500	-0.00025300
H	-2.96548400	2.77052200	-0.00060600
C	-1.71201700	0.19967500	-0.00021000
S	-3.14586700	-2.04243000	0.00032500
C	-0.57084000	0.99296000	-0.00040500
H	-0.63520100	2.07435500	-0.00057300
O	1.68097600	2.58449900	-0.00034000

TX-DBT Triplet

C	5.93949700	-0.11342000	-0.00034300
C	4.90060000	-1.03659000	-0.00014200
C	3.57093500	-0.59320000	-0.00000900
C	3.23117600	0.79820100	-0.00007300
C	4.31989400	1.70643100	-0.00022800
C	5.63109000	1.26256900	-0.00038400
C	1.87836700	1.30531400	0.00010100
C	0.72347900	0.43180300	0.00021700
C	0.80331000	-1.00824000	0.00014200
C	-0.33371000	-1.82507800	0.00000200
H	-0.22912800	-2.90644800	0.00005900
C	-1.58610500	-1.22429400	-0.00007000
H	6.97096000	-0.45027700	-0.00052300
H	5.11226900	-2.10305500	0.00000100

H	4.07758700	2.76254400	-0.00022200
H	6.43859700	1.99037300	-0.00052600
S	2.33867800	-1.84228200	0.00052900
C	-3.09933200	0.60972200	0.00008300
C	-3.98983200	-0.49046300	-0.00017700
C	-5.37176000	-0.30725200	-0.00030200
C	-5.86916500	0.99578900	-0.00012700
C	-5.00172800	2.09979400	0.00015300
C	-3.62501200	1.91283900	0.00025500
H	-6.04757300	-1.15723300	-0.00051500
H	-6.94381900	1.15516600	-0.00020600
H	-5.41031900	3.10589500	0.00028400
H	-2.95330800	2.76636900	0.00045100
C	-1.71010300	0.19709500	0.00009800
S	-3.14760700	-2.04771400	-0.00028600
C	-0.56729800	0.99676700	0.00023000
H	-0.62847500	2.07878600	0.00031300
O	1.68628100	2.58638600	0.00005600

BDBT Singlet

C	-4.64809200	2.14263800	-0.45721900
C	-5.58825600	1.10294400	-0.39284900
C	-5.18052500	-0.21243300	-0.18201300
C	-3.81639300	-0.46975600	-0.03703100
C	-2.85456200	0.56351800	-0.09921600
C	-3.28989000	1.87945500	-0.31179600
H	-4.98389600	3.16239300	-0.62188300
H	-6.64584000	1.32292300	-0.50796300
H	-5.90801000	-1.01725200	-0.13161400
H	-2.56729000	2.68935100	-0.36168200
C	-1.47561700	-1.34015700	0.27135000
C	-0.26209200	-2.02517900	0.47454800
C	0.92205300	-1.31621000	0.47818700
C	0.94421700	0.09433700	0.24276500
C	-0.29866100	0.77177300	0.07025800
C	-1.49768800	0.06274900	0.07966500
H	-0.25588300	-3.09792000	0.64493500
H	1.85369200	-1.83302700	0.67988800
H	-0.28991400	1.84261900	-0.09665600
S	-3.07967200	-2.05590500	0.23512200
C	2.16555500	0.85537300	0.27718200
C	3.51258200	0.41106800	0.01746300
C	4.61184600	1.13295900	0.55898500
C	3.79379700	-0.69388600	-0.82943100
C	5.91635300	0.73676400	0.29826800
H	4.41401600	1.98397400	1.20129100
C	5.10315300	-1.08391300	-1.07094400
H	2.97633800	-1.22110200	-1.31067600
C	6.17646300	-0.37641600	-0.50997900

H	6.74110100	1.29403000	0.73505100
H	5.29523700	-1.93652500	-1.71726300
H	7.19885100	-0.68399500	-0.70927700
O	2.00511200	2.12190400	0.64559300

BDBT Triplet

C	-4.63578900	2.15336400	-0.46804300
C	-5.58181700	1.11889900	-0.40223700
C	-5.18165300	-0.19783900	-0.18776300
C	-3.81865400	-0.46196000	-0.04062600
C	-2.85119100	0.56601400	-0.10408200
C	-3.27913100	1.88360500	-0.32027400
H	-4.96600300	3.17444500	-0.63569500
H	-6.63799700	1.34453800	-0.51905400
H	-5.91336800	-0.99870400	-0.13600900
H	-2.55234400	2.68965700	-0.37115900
C	-1.48274600	-1.34331900	0.27397300
C	-0.27520000	-2.03467500	0.47840800
C	0.91357400	-1.33128500	0.48414300
C	0.94048500	0.07514500	0.24652100
C	-0.29297200	0.75867500	0.06760500
C	-1.49763000	0.05771600	0.07816700
H	-0.27469900	-3.10794000	0.64580000
H	1.84312000	-1.85253500	0.68413800
H	-0.27621800	1.82887100	-0.10518700
S	-3.09081600	-2.05042100	0.23507200
C	2.16776000	0.83860900	0.29357500
C	3.51890600	0.40352600	0.02084500
C	4.61153200	1.13322300	0.55815300
C	3.80170100	-0.69490900	-0.82927700
C	5.91912200	0.75395600	0.28348600
H	4.40838900	1.97865900	1.20724500
C	5.11422400	-1.07028700	-1.08338200
H	2.98562000	-1.23176700	-1.30231700
C	6.18367500	-0.35258700	-0.53092900
H	6.74134700	1.31951300	0.71444700
H	5.30952300	-1.92041500	-1.73211100
H	7.20765100	-0.64848000	-0.74007800
O	1.99160800	2.08695700	0.70240700

FBDBT Singlet

C	-5.06055300	2.07493400	-0.52963900
C	-5.98068700	1.01747400	-0.46576100
C	-5.55021600	-0.28663300	-0.23125800
C	-4.18361100	-0.51432600	-0.06211000
C	-3.24175200	0.53715200	-0.12322500
C	-3.69973100	1.84115100	-0.36000500
H	-5.41411400	3.08543600	-0.71305800

H	-7.04050900	1.21471700	-0.59981000
H	-6.26220400	-1.10519200	-0.18108800
H	-2.99275600	2.66471700	-0.40970900
C	-1.83152500	-1.33363700	0.29408900
C	-0.60746700	-1.99153300	0.52629900
C	0.56199700	-1.25941900	0.53847800
C	0.56078500	0.14886800	0.28360000
C	-0.69394700	0.79914500	0.08282000
C	-1.87805100	0.06624600	0.08326200
H	-0.58300900	-3.06136100	0.71267000
H	1.49955700	-1.75538200	0.76423600
H	-0.70344700	1.86766700	-0.09782500
S	-3.41967100	-2.08173100	0.24249300
C	1.76391700	0.93456600	0.32388400
C	3.12489000	0.51047500	0.10003800
C	4.19901200	1.26162700	0.65258800
C	3.44633700	-0.59988700	-0.72570800
C	5.51832600	0.89537600	0.42962000
H	3.97249100	2.11927500	1.27561200
C	4.76468800	-0.97461400	-0.93835600
H	2.65134100	-1.15098300	-1.21684300
C	5.78706700	-0.22363800	-0.35634300
H	6.34174400	1.45470300	0.86199200
H	5.01714700	-1.82446300	-1.56430500
O	1.57676300	2.20662300	0.66170800
F	7.06861300	-0.58604200	-0.57100800

FBDBT Triplet

C	-5.04757000	2.08787900	-0.54116100
C	-5.97440500	1.03617700	-0.47621500
C	-5.55243000	-0.26964400	-0.23796900
C	-4.18706800	-0.50510000	-0.06618000
C	-3.23869700	0.54058000	-0.12833600
C	-3.68838500	1.84657400	-0.36875600
H	-5.39471400	3.10004000	-0.72758000
H	-7.03274700	1.23968400	-0.61238800
H	-6.26932000	-1.08383100	-0.18682000
H	-2.97659000	2.66591900	-0.41914500
C	-1.84020000	-1.33688200	0.29671200
C	-0.62277100	-2.00200900	0.52986800
C	0.55193500	-1.27596300	0.54449100
C	0.55636900	0.12804900	0.28824600
C	-0.68801800	0.78518500	0.08057600
C	-1.87852500	0.06118000	0.08188400
H	-0.60459600	-3.07260500	0.71268800
H	1.48726100	-1.77718900	0.76810300
H	-0.68863300	1.85313300	-0.10621200
S	-3.43322300	-2.07532200	0.24213000
C	1.76573700	0.91516300	0.34166100

C	3.13075000	0.49846900	0.10762800
C	4.19861900	1.25298300	0.66158400
C	3.45389800	-0.60248200	-0.72559000
C	5.52085200	0.90148400	0.42665100
H	3.96700700	2.10258600	1.29470700
C	4.77502500	-0.96490000	-0.94835200
H	2.66023900	-1.15909700	-1.21286500
C	5.79387700	-0.20844500	-0.36896600
H	6.34181100	1.46521700	0.85800300
H	5.03074200	-1.81005100	-1.57937100
O	1.56746700	2.17256500	0.71197900
F	7.07749100	-0.55877400	-0.59396600

ClBDBT Singlet

C	-5.46030800	2.01151200	-0.62602400
C	-6.36443600	0.94021700	-0.56393700
C	-5.91840800	-0.35260400	-0.29837500
C	-4.55222500	-0.55465900	-0.09633900
C	-3.62641100	0.51101200	-0.15503800
C	-4.09986100	1.80325300	-0.42351400
H	-5.82610700	3.01281700	-0.83402200
H	-7.42411100	1.11779200	-0.72388700
H	-6.61805100	-1.18177900	-0.24960000
H	-3.40549300	2.63752000	-0.47218500
C	-2.19567900	-1.32976000	0.32369200
C	-0.96772100	-1.96413800	0.59338400
C	0.18967300	-1.21228000	0.61895500
C	0.16974600	0.18944900	0.34034900
C	-1.08764200	0.81659100	0.10245200
C	-2.26052700	0.06545100	0.08852500
H	-0.93040100	-3.03028600	0.79746300
H	1.13028200	-1.68852600	0.87263900
H	-1.10934900	1.88224500	-0.09367500
S	-3.76970000	-2.10395700	0.25072900
C	1.36221000	0.99821900	0.39511900
C	2.72748900	0.59994700	0.18156600
C	3.78713600	1.38513900	0.71709900
C	3.07692300	-0.52159000	-0.61912400
C	5.11288300	1.04167000	0.50402700
H	3.54488300	2.25101900	1.32281100
C	4.40226200	-0.87033500	-0.82317800
H	2.29718000	-1.10229300	-1.10069700
C	5.42042900	-0.09030900	-0.25853900
H	5.91135800	1.63815200	0.93300100
H	4.65639100	-1.73114700	-1.43291400
O	1.14091500	2.26257700	0.73597400
Cl	7.09812400	-0.53197200	-0.52500000

ClBDBT Triplet

C	-5.44208000	2.02824700	-0.64776600
C	-6.35457000	0.96379500	-0.58688900
C	-5.91954500	-0.33113300	-0.31521400
C	-4.55547500	-0.54233500	-0.10588300
C	-3.62149900	0.51637800	-0.16327500
C	-4.08412900	1.81102700	-0.43785300
H	-5.79954600	3.03151600	-0.86063100
H	-7.41204300	1.14883400	-0.75260800
H	-6.62530400	-1.15512700	-0.26720100
H	-3.38368900	2.64023800	-0.48542500
C	-2.20671400	-1.33270200	0.32916700
C	-0.98658200	-1.97598700	0.60444800
C	0.17696600	-1.23197900	0.63584800
C	0.16604500	0.16569500	0.35354600
C	-1.08038000	0.80166900	0.10504500
C	-2.26041900	0.06082400	0.08877100
H	-0.95779300	-3.04293900	0.80589300
H	1.11398200	-1.71448200	0.89105500
H	-1.09100000	1.86656300	-0.09779500
S	-3.78602800	-2.09523400	0.24853200
C	1.36537100	0.97469700	0.42479700
C	2.73382900	0.58358900	0.19629500
C	3.78795400	1.37361900	0.72736000
C	3.08195300	-0.53129500	-0.60851800
C	5.11588100	1.04461200	0.49848100
H	3.54228100	2.23405700	1.34067000
C	4.40953400	-0.86790400	-0.82724700
H	2.30231800	-1.11969700	-1.08108300
C	5.42494200	-0.08036500	-0.27165900
H	5.91302400	1.64729700	0.92124900
H	4.66485600	-1.72591600	-1.44045000
O	1.12713900	2.21731100	0.81458500
Cl	7.10504500	-0.50634200	-0.55668100

BrBDBT Singlet

C	-6.21050800	1.93311600	-0.71682700
C	-7.09382500	0.84468500	-0.65314600
C	-6.62690300	-0.43403300	-0.35701700
C	-5.26100200	-0.60459300	-0.12606700
C	-4.35596800	0.47871600	-0.18585700
C	-4.85024100	1.75626400	-0.48537800
H	-6.59243300	2.92304700	-0.94889300
H	-8.15363700	0.99774700	-0.83590200
H	-7.31045600	-1.27644900	-0.30700800
H	-4.17205900	2.60365600	-0.53552300
C	-2.89758500	-1.32403400	0.35075800
C	-1.66252200	-1.92843100	0.65386200
C	-0.52120500	-1.15251100	0.68786700

C	-0.56440900	0.24345800	0.38514900
C	-1.82914500	0.84055600	0.11350600
C	-2.98622800	0.06539700	0.09060800
H	-1.60744700	-2.99001300	0.87682500
H	0.42394500	-1.60501000	0.96690900
H	-1.86873500	1.90217800	-0.10093600
S	-4.45409700	-2.13147300	0.26203300
C	0.61064700	1.07739800	0.44804700
C	1.98679600	0.70410000	0.26386000
C	3.02117800	1.52074700	0.80270300
C	2.37257100	-0.42387500	-0.51128000
C	4.35699100	1.20084700	0.61727800
H	2.75133400	2.39182300	1.38906800
C	3.70820200	-0.74769800	-0.68749600
H	1.61332500	-1.02929800	-0.99525000
C	4.69992500	0.06289300	-0.11995200
H	5.13481100	1.82182900	1.04881300
H	3.98907000	-1.61378900	-1.27726500
O	0.35850400	2.34258900	0.76314900
Br	6.53740500	-0.38402500	-0.37149300

BrBDBT Triplet

C	-6.19047000	1.95231500	-0.74290200
C	-7.08324300	0.87132700	-0.68104600
C	-6.62878900	-0.40985000	-0.37781300
C	-5.26536000	-0.59049100	-0.13794400
C	-4.35107300	0.48524200	-0.19578200
C	-4.83305900	1.76555800	-0.50243400
H	-6.56292500	2.94448600	-0.98063000
H	-8.14056100	1.03259200	-0.87084800
H	-7.31930400	-1.24661700	-0.32904300
H	-4.14795900	2.60743200	-0.55101100
C	-2.91086100	-1.32684300	0.35691700
C	-1.68435400	-1.94109100	0.66738300
C	-0.53623900	-1.17376800	0.70829100
C	-0.56872800	0.21817700	0.40123800
C	-1.82178400	0.82510400	0.11737600
C	-2.98675200	0.06099600	0.09092600
H	-1.63890900	-3.00360600	0.88825400
H	0.40471500	-1.63321400	0.99019800
H	-1.84898000	1.88600500	-0.10404800
S	-4.47328000	-2.12158300	0.25894600
C	0.61347500	1.05176500	0.48147200
C	1.99283000	0.68387900	0.28409500
C	3.02177200	1.50431600	0.81922800
C	2.37698200	-0.43816000	-0.49444500
C	4.35968700	1.19722400	0.61955600
H	2.74858500	2.37050700	1.41254500
C	3.71477000	-0.75153700	-0.68376200

H	1.61779000	-1.05050000	-0.96997000
C	4.70378600	0.06555300	-0.12449600
H	5.13627700	1.82358000	1.04553400
H	3.99669000	-1.61538000	-1.27634900
O	0.34480300	2.29613000	0.84430500
Br	6.54380300	-0.36678000	-0.39392400

XA Singlet

C	-0.96562100	-0.00002600	3.59822200
C	-1.60002800	0.00002800	2.35147100
C	-0.83868100	0.00011200	1.19188400
C	0.57860700	-0.00001300	1.24011900
C	1.19841700	0.00003200	2.51319800
C	0.43116100	0.00004900	3.67173800
C	1.28507000	-0.00011800	0.00000000
C	0.57860700	-0.00001300	-1.24011900
C	-0.83868100	0.00011200	-1.19188400
C	-1.60002800	0.00002800	-2.35147100
H	-2.68146900	-0.00005400	-2.26160400
C	-0.96562100	-0.00002600	-3.59822200
C	0.43116100	0.00004900	-3.67173800
C	1.19841700	0.00003200	-2.51319800
H	-1.56359400	-0.00007500	4.50422500
H	-2.68146900	-0.00005400	2.26160400
H	2.28117600	-0.00000400	2.57625200
H	0.92468200	0.00011600	4.63928400
H	-1.56359400	-0.00007500	-4.50422500
H	0.92468200	0.00011600	-4.63928400
H	2.28117600	-0.00000400	-2.57625200
O	2.61044100	-0.00003800	0.00000000
O	-1.52022500	-0.00014200	0.00000000

XA Triplet

C	-0.96398600	-0.00023600	3.60055000
C	-1.60019800	-0.00006000	2.35425600
C	-0.84067400	0.00015100	1.19329000
C	0.57562300	0.00000500	1.24385000
C	1.19837400	-0.00006300	2.51353800
C	0.43283200	-0.00017900	3.67376900
C	1.28553200	-0.00006500	0.00000000
C	0.57562300	0.00000500	-1.24385000
C	-0.84067400	0.00015100	-1.19329000
C	-1.60019800	-0.00006000	-2.35425600
H	-2.68193100	-0.00009600	-2.26651000
C	-0.96398600	-0.00023600	-3.60055000
C	0.43283200	-0.00017900	-3.67376900
C	1.19837400	-0.00006300	-2.51353800
H	-1.56138700	-0.00039600	4.50703800

H	-2.68193100	-0.00009600	2.26651000
H	2.28243700	-0.00003200	2.56858100
H	0.92722000	-0.00018900	4.64091200
H	-1.56138700	-0.00039600	-4.50703800
H	0.92722000	-0.00018900	-4.64091200
H	2.28243700	-0.00003200	-2.56858100
O	2.61458100	0.00025400	0.00000000
O	-1.52327100	0.00054500	0.00000000

AR Singlet

C	-0.94577400	0.00018400	3.64563100
C	-1.60405200	0.00001100	2.41453200
C	-0.85690100	-0.00014500	1.23311000
C	0.57766100	-0.00008800	1.24294200
C	1.21054600	0.00009900	2.50471700
C	0.45985100	0.00015900	3.67520300
C	1.30177900	-0.00020400	0.00000000
C	0.57766100	-0.00008800	-1.24294200
C	-0.85690100	-0.00014500	-1.23311000
C	-1.60405200	0.00001100	-2.41453200
H	-2.69095400	0.00021700	-2.36422800
C	-0.94577400	0.00018400	-3.64563100
C	0.45985100	0.00015900	-3.67520300
C	1.21054600	0.00009900	-2.50471700
H	-1.51691600	-0.00007600	4.56808800
H	-2.69095400	0.00021700	2.36422800
H	2.29406600	0.00033600	2.52570900
H	0.97177700	0.00029700	4.63416300
H	-1.51691600	-0.00007600	-4.56808800
H	0.97177700	0.00029700	-4.63416300
H	2.29406600	0.00033600	-2.52570900
O	2.61018200	-0.00016200	0.00000000
N	-1.48620800	-0.00017000	0.00000000
H	-2.50059600	-0.00045700	0.00000000

AR Triplet

C	-0.91529000	-0.00030800	3.65278100
C	-1.58469600	-0.00004600	2.42437800
C	-0.86626000	0.00019800	1.22854300
C	0.55869600	0.00009800	1.25435600
C	1.21363300	-0.00014400	2.50713200
C	0.48197300	-0.00031500	3.69014800
C	1.24965800	0.00020700	0.00000000
C	0.55869600	0.00009800	-1.25435600
C	-0.86626000	0.00019800	-1.22854300
C	-1.58469600	-0.00004600	-2.42437800
H	-2.67211700	-0.00001400	-2.39062700

C	-0.91529000	-0.00030800	-3.65278100
C	0.48197300	-0.00031500	-3.69014800
C	1.21363300	-0.00014400	-2.50713200
H	-1.48949700	-0.00053500	4.57440200
H	-2.67211700	-0.00001400	2.39062700
H	2.29888700	-0.00022000	2.53230800
H	1.00236600	-0.00049400	4.64353000
H	-1.48949700	-0.00053500	-4.57440200
H	1.00236600	-0.00049400	-4.64353000
H	2.29888700	-0.00022000	-2.53230800
O	2.58310100	0.00034500	0.00000000
N	-1.51103800	0.00065300	0.00000000
H	-2.52144200	0.00015000	0.00000000

AD Singlet

C	-0.62853300	0.00018900	3.81586500
C	-1.39993100	0.00012500	2.65827500
C	-0.78508900	-0.00006900	1.40274500
C	0.64342600	-0.00008200	1.26733900
C	1.39739600	-0.00003200	2.47532900
C	0.77734200	0.00008100	3.70881500
C	1.30629600	-0.00003700	0.00000000
C	0.64342600	-0.00008200	-1.26733900
C	-0.78508900	-0.00006900	-1.40274500
C	-1.39993100	0.00012500	-2.65827500
H	-2.48504900	0.00028800	-2.72368800
C	-0.62853300	0.00018900	-3.81586500
C	0.77734200	0.00008100	-3.70881500
C	1.39739600	-0.00003200	-2.47532900
H	-1.10782300	0.00021900	4.78963400
H	-2.48504900	0.00028800	2.72368800
H	2.47701600	-0.00006300	2.38958700
H	1.38385500	0.00009500	4.61062000
H	-1.10782300	0.00021900	-4.78963400
H	1.38385500	0.00009500	-4.61062000
H	2.47701600	-0.00006300	-2.38958700
O	2.65100500	-0.00002100	0.00000000
S	-1.85232100	-0.00020300	0.00000000

AD Triplet

C	-0.64375900	-0.00026000	3.81252200
C	-1.40780700	-0.00016800	2.65249200
C	-0.77873600	-0.00000300	1.39924000
C	0.64977200	0.00003200	1.26563200
C	1.39190800	-0.00001500	2.47407400
C	0.76295500	-0.00017000	3.70656400
C	1.34226700	0.00014900	0.00000000
C	0.64977200	0.00003200	-1.26563200

C	-0.77873600	-0.00000300	-1.39924000
C	-1.40780700	-0.00016800	-2.65249200
H	-2.49350100	-0.00016100	-2.70860200
C	-0.64375900	-0.00026000	-3.81252200
C	0.76295500	-0.00017000	-3.70656400
C	1.39190800	-0.00001500	-2.47407400
H	-1.12530700	-0.00044600	4.78489200
H	-2.49350100	-0.00016100	2.70860200
H	2.47189600	0.00006800	2.38791600
H	1.36590200	-0.00021900	4.61103000
H	-1.12530700	-0.00044600	-4.78489200
H	1.36590200	-0.00021900	-4.61103000
H	2.47189600	0.00006800	-2.38791600
O	2.64545500	0.00022100	0.00000000
S	-1.83420100	0.00036600	0.00000000

Table S2: The geometry optimization with TDDFT and UDFT for HA are singlet and triplet, respectively. ΔE_1 and ΔE_2 (in ev) are the energy difference between the first excited triplet states and the second excited triplet states obtained by TDDFT and the ground states. The ground states and excited states energy calculated by UDFT are E_s and E_t (in a.u.).

		Singlet				Triplet			
		ΔE_1	ΔE_2	E_s	E_t	ΔE_1	ΔE_2	E_s	E_t
PBE	cc-pvdz	1.4564	1.6708*	-763.1686	-763.0374	1.4651	1.6702*	-763.1690	-763.0823
	cc-pvtz	1.4720	1.6711*	-763.3662	-763.2298	1.4801	1.6707*	-763.3664	-763.2189
	aug-cc-pvdz	1.4654	1.6762*	-763.2114	-763.0936	1.4735	1.6757*	-763.2117	-763.0920
	aug-cc-pvtz	1.4698	1.6738*	-763.3761	-763.2941	1.4777	1.6734*	-763.3763	-763.0920
PBE0	cc-pvdz	1.7490	1.9556	-763.2029	-763.1277	1.7501	1.9626	-763.2030	-763.1277
	cc-pvtz	1.7453	1.9692	-763.3881	-763.3141	1.7467	1.9759	-763.3882	-763.3125
	aug-cc-pvdz	1.7632	1.9782	-763.2395	-763.1647	1.7647	1.9850	-763.2396	-763.1647
	aug-cc-pvtz	1.7476	1.9729	-763.3961	-763.3208	1.7491	1.9794	-763.3962	-763.3208
B3LYP	cc-pvdz	1.7966	1.9342	-764.0532	-763.9763	1.7972	1.9411	-764.0534	-763.9765
	cc-pvtz	1.7968	1.9574	-764.2647	-764.1886	1.7976	1.9639	-764.2648	-764.1886
	aug-cc-pvdz	1.8035	1.9576	-764.0955	-764.0194	1.8044	1.9642	-764.0958	-764.0196
	aug-cc-pvtz	1.7970	1.9611	-764.2736	-764.1978	1.7979	1.9675	-764.2737	-764.1978
CAM-B3LYP	cc-pvdz	1.8504	2.2322	-763.6719	-763.5906	1.8528	2.2378	-763.6721	-763.5907
	cc-pvtz	1.8642	2.2635	-763.8871	-763.8068	1.8669	2.2688	-763.8871	-763.8068

	aug-cc-pvdz	1.8776	2.2664	-763.7156	-763.6355	1.8805	2.2718	-763.7157	-763.6356
	aug-cc-pvtz	1.8691	2.2691	-763.8961	-763.8163	1.8720	2.2743	-763.8961	-763.8163
WB97XD	cc-pvdz	1.9751	2.2580	-763.7979	-763.7145	1.9773	2.2640	-763.7980	-763.7146
	cc-pvtz	1.9709	2.2757	-763.9885	-763.9064	1.9735	2.2814	-763.9885	-763.9064
	aug-cc-pvdz	1.9900	2.2863	-763.8332	-763.7532	1.9926	2.2921	-763.8334	-763.7532
	aug-cc-pvtz	1.9736	2.2804	-763.9967	-763.9150	1.9762	2.2861	-763.9968	-763.9150
M062X	cc-pvdz	2.1550	2.2099	-763.7836	-763.6971	2.1553	2.2143	-763.7838	-763.6973
	cc-pvtz	2.1313	2.2836	-763.9675	-763.8823	2.1320	2.2877	-763.9675	-763.8824
	aug-cc-pvdz	2.1536	2.2412	-763.8211	-763.7360	2.1544	2.2455	-763.8213	-763.7361
	aug-cc-pvtz	2.1283	2.2927	-763.9761	-763.8961	2.1292	2.2967	-763.9762	-763.8960
M06HF	cc-pvdz	1.9965	2.4240*	-763.8777	-763.7859	1.9990	2.4227*	-763.8778	-763.7860
	cc-pvtz	2.1305	2.3006*	-764.1273	-764.0372	2.1330	2.2996*	-764.1274	-764.0372
	aug-cc-pvdz	2.0641	2.4180*	-763.9265	-763.8365	2.0668	2.4179*	-763.9266	-763.8367
	aug-cc-pvtz	2.1441	2.2939*	-764.1478	-764.0583	2.1465	2.2934*	-764.1478	-764.0584

Table S3: The geometry optimization with TDDFT and UDFT for AAT are singlet and triplet, respectively. ΔE_1 and ΔE_2 (in ev) are the energy difference between the first excited triplet states and the second excited triplet states obtained by TDDFT and the ground states. The ground states and excited states energy calculated by UDFT are E_s and E_t (in a.u.).

		Singlet				Triplet			

		ΔE_1	ΔE_2	E_s	E_t	ΔE_1	ΔE_2	E_s	E_t
PBE	cc-pvdz	1.2479	1.7396	-743.3080	-743.2495	1.2778	1.7606	-743.3089	-743.2499
	cc-pvtz	1.2515	1.7452	-743.4994	-743.4398	1.2808	1.7657	-743.5001	-743.4403
	aug-cc-pvdz	1.2553	1.7343	-743.3500	-743.2920	1.2845	1.7545	-743.3507	-743.2924
	aug-cc-pvtz	1.2549	1.7370	-743.5093	-743.4500	1.2840	1.7571	-743.5100	-743.4505
PBE0	cc-pvdz	1.3515	2.2710	-743.3476	-743.2874	1.3696	2.2940	-743.3484	-743.2880
	cc-pvtz	1.3431	2.2709	-743.5263	-743.4672	1.3611	2.2933	-743.5271	-743.4675
	aug-cc-pvdz	1.3488	2.2736	-743.3831	-743.3240	1.3669	2.2956	-743.3838	-743.3244
	aug-cc-pvtz	1.3445	2.2672	-743.5343	-743.4755	1.3625	2.2891	-743.5351	-743.4758
B3LYP	cc-pvdz	1.3689	2.2447	-744.1824	-744.1210	1.3898	2.2676	-744.1831	-744.1215
	cc-pvtz	1.3647	2.2564	-744.3883	-744.3278	1.3856	2.2787	-744.3889	-744.3281
	aug-cc-pvdz	1.3684	2.2491	-744.2243	-744.1640	1.3894	2.2710	-744.2249	-744.1643
	aug-cc-pvtz	1.3672	2.2524	-744.3972	-744.3370	1.3880	2.2742	-744.3978	-744.3373
CAM-B3LYP	cc-pvdz	1.5111	2.5246	-743.7981	-743.7354	1.5145	2.5329	-743.7989	-743.7358
	cc-pvtz	1.4984	2.5458	-744.0074	-743.9458	1.5021	2.5508	-744.0080	-743.9461
	aug-cc-pvdz	1.4995	2.5545	-743.8410	-743.7797	1.5038	2.5591	-743.8417	-743.7799
	aug-cc-pvtz	1.4981	2.5505	-744.0164	-743.9552	1.5022	2.5534	-744.0170	-743.9554
WB97XD	cc-pvdz	1.5828	2.5652	-743.9335	-743.8692	1.5845	2.5880	-743.9342	-743.8696
	cc-pvtz	1.5717	2.5688	-744.1175	-744.0544	1.5735	2.5910	-744.1181	-744.0546
	aug-cc-pvdz	1.5761	2.5713	-743.9679	-743.9049	1.5783	2.5930	-743.9685	-743.9051

	aug-cc-pvtz	1.5732	2.5657	-744.1257	-744.0629	1.5751	2.5873	-744.1264	-744.0631
M062X	cc-pvdz	1.7111	2.5195	-743.9150	-743.8482	1.7075	2.5418	-743.9157	-743.8486
	cc-pvtz	1.6969	2.5750	-744.0928	-744.0272	1.6951	2.5965	-744.0934	-744.0275
	aug-cc-pvdz	1.7005	2.5279	-743.9521	-743.8865	1.6986	2.5491	-743.9528	-743.8868
	aug-cc-pvtz	1.6951	2.5750	-744.1016	-744.0364	1.6939	2.5961	-744.1022	-744.0366
M06HF	cc-pvdz	1.9883	2.2822	-744.0116	-743.9422	1.9456	2.3025	-744.0125	-743.9429
	cc-pvtz	1.9501	2.3892	-744.2549	-744.1872	1.9172	2.4084	-744.2557	-744.1875
	aug-cc-pvdz	1.9663	2.3187	-744.0606	-743.9926	1.9302	2.3377	-744.0614	-743.9930
	aug-cc-pvtz	1.9387	2.3949	-744.2760	-744.2086	1.9090	2.4137	-744.2768	-744.2089

Table S4: The geometry optimization with TDDFT and UDFT for BP are singlet and triplet, respectively. ΔE_1 and ΔE_2 (in ev) are the energy difference between the first excited triplet states and the second excited triplet states obtained by TDDFT and the ground states. The ground states and excited states energy calculated by UDFT are E_s and E_t (in a.u.).

		Singlet				Triplet			
		ΔE_1	ΔE_2	E_s	E_t	ΔE_1	ΔE_2	E_s	E_t
PBE	cc-pvdz	1.8108	2.6989	-575.9319	-575.8497	1.8453	2.7454	-575.9330	-575.8497
	cc-pvtz	1.8441	2.6861	-576.0823	-576.0000	1.8776	2.7317	-576.0834	-576.0002
	aug-cc-pvdz	1.8498	2.6941	-575.9622	-575.8794	1.8827	2.7389	-575.9632	-575.8795
	aug-cc-pvtz	1.8527	2.6812	-576.0891	-576.0068	1.8856	2.7261	-576.0901	-576.0069

PBE0	cc-pvdz	2.0304	2.5229	-575.9805	-575.8996	2.0712	2.5691	-575.9819	-575.8998
	cc-pvtz	2.0692	2.5309	-576.1193	-576.0387	2.1091	2.5753	-576.1207	-576.0389
	aug-cc-pvdz	2.1005	2.5556	-576.0052	-575.9235	2.1388	2.5998	-576.0065	-575.9237
	aug-cc-pvtz	2.0906	2.5373	-576.1246	-576.0439	2.1296	2.5809	-576.1260	-576.0442
B3LYP	cc-pvdz	2.0767	2.6019	-576.6506	-576.5668	2.1136	2.6505	-576.6519	-576.5670
	cc-pvtz	2.1252	2.6187	-576.8142	-576.7301	2.1610	2.6658	-576.8155	-576.7303
	aug-cc-pvdz	2.1451	2.6323	-576.6810	-576.5963	2.1798	2.6789	-576.6822	-576.5964
	aug-cc-pvtz	2.1461	2.6235	-576.8202	-576.7361	2.1811	2.6699	-576.8215	-576.7362
CAM-B3LYP	cc-pvdz	2.1285	2.6329	-576.3248	-576.2401	2.1758	2.6720	-576.3263	-576.2403
	cc-pvtz	2.186	2.6696	-576.4908	-576.4058	2.2321	2.7066	-576.4924	-576.4061
	aug-cc-pvdz	2.2143	2.6819	-576.3558	-576.2700	2.2588	2.7183	-576.3572	-576.2702
	aug-cc-pvtz	2.2112	2.679	-576.4969	-576.4118	2.2563	2.7150	-576.4984	-576.4121
WB97XD	cc-pvdz	2.2408	2.7201	-576.4533	-576.3662	2.2829	2.7639	-576.4547	-576.3664
	cc-pvtz	2.2793	2.7324	-576.5977	-576.5109	2.3210	2.7739	-576.5992	-576.5113
	aug-cc-pvdz	2.3111	2.7551	-576.4776	-576.3897	2.3511	2.7964	-576.4790	-576.3899
	aug-cc-pvtz	2.2998	2.7391	-576.6033	-576.5165	2.3408	2.7797	-576.6048	-576.5168
M062X	cc-pvdz	2.2744	2.794	-576.4281	-576.3393	2.3070	2.8495	-576.4297	-576.3395
	cc-pvtz	2.3525	2.8028	-576.5681	-576.4792	2.3871	2.8529	-576.5696	-576.4795
	aug-cc-pvdz	2.3379	2.8169	-576.4547	-576.3654	2.3692	2.8695	-576.4562	-576.3656
	aug-cc-pvtz	2.3728	2.8071	-576.5739	-576.4851	2.4070	2.8560	-576.5754	-576.4854

M06HF	cc-pvdz	2.0735	2.853	-576.5183	-576.4280	2.0901	2.9220	-576.5201	-576.4283
	cc-pvtz	2.2186	2.7516	-576.7094	-576.6197	2.2378	2.8134	-576.7112	-576.6200
	aug-cc-pvdz	2.1733	2.8704	-576.5523	-576.4610	2.1883	2.9355	-576.5540	-576.4613
	aug-cc-pvtz	2.2402	2.7536	-576.7249	-576.6355	2.2590	2.8141	-576.7266	-576.6358

Table S5: The geometry optimization with TDDFT and UDFT for TX-BT are singlet and triplet, respectively. ΔE_1 and ΔE_2 (in ev) are the energy difference between the first excited triplet states and the second excited triplet states obtained by TDDFT and the ground states. The ground states and excited states energy calculated by UDFT are E_s and E_t (in a.u.).

		Singlet				Triplet			
		ΔE_1	ΔE_2	E_s	E_t	ΔE_1	ΔE_2	E_s	E_t
PBE	cc-pvdz	2.7426	2.8744	-1372.9808	-1372.8736	2.2143	3.2628	-1372.9774	-1372.8910
	cc-pvtz	2.7060	2.8212	-1373.1564	-1373.0508	2.1840	3.1924	-1373.1537	-1373.0686
	aug-cc-pvdz	2.7131	2.8254	-1373.0133	-1372.9075	2.1945	3.1635	-1373.0101	-1372.9247
	aug-cc-pvtz	2.6936	2.7992	-1373.1635	-1373.0585	2.1770	3.1434	-1373.1608	-1373.0760
PBE0	cc-pvdz	2.6963	2.7337	-1373.1021	-1372.9881	1.9897	3.3536	-1373.0981	-1373.0112
	cc-pvtz	2.6574	2.6942	-1373.2666	-1373.1542	1.9619	3.3042	-1373.2633	-1373.1776
	aug-cc-pvdz	2.6780	2.7098	-1373.1288	-1373.0162	1.9815	3.3189	-1373.1251	-1373.0390
	aug-cc-pvtz	2.6508	2.6849	-1373.2720	-1373.1602	1.9598	3.2891	-1373.2687	-1373.1833
B3LYP	cc-pvdz	2.7929	2.8430	-1374.0702	-1373.9564	2.1036	3.4602	-1374.0664	-1373.9788

	cc-pvtz	2.7636	2.8062	-1374.2591	-1374.1469	2.0843	3.4103	-1374.2560	-1374.1695
	aug-cc-pvdz	2.7733	2.8136	-1374.1022	-1373.9899	2.0961	3.4059	-1374.0987	-1374.0119
	aug-cc-pvtz	2.7556	2.7933	-1374.2653	-1374.1536	2.0814	3.3832	-1374.2622	-1374.1760
CAM-B3LYP	cc-pvdz	2.6453	2.6946	-1373.7163	-1373.5968	1.8539	3.3422	-1373.7116	-1373.6232
	cc-pvtz	2.6200	2.6735	-1373.9085	-1373.7905	1.8421	3.3151	-1373.9045	-1373.8172
	aug-cc-pvdz	2.6329	2.6809	-1373.7487	-1373.6306	1.8556	3.3216	-1373.7442	-1373.6567
	aug-cc-pvtz	2.6154	2.6679	-1373.9147	-1373.7973	1.8434	3.3055	-1373.9107	-1373.8238
WB97XD	cc-pvdz	2.8124	2.8442	-1373.8425	-1373.7205	2.0122	3.5241	-1373.8374	-1373.7466
	cc-pvtz	2.7768	2.8153	-1374.0106	-1373.8901	1.9922	3.4827	-1374.0061	-1373.9165
	aug-cc-pvdz	2.7957	2.8285	-1373.8685	-1373.7479	2.0094	3.4990	-1373.8636	-1373.7736
	aug-cc-pvtz	2.7706	2.8092	-1374.0163	-1373.8963	1.9916	3.4719	-1374.0119	-1373.9225
M062X	cc-pvdz	3.2429	3.2730	-1373.7953	-1373.6668	2.4672	3.9289	-1373.7902	-1373.6926
	cc-pvtz	3.1623	3.1886	-1373.9653	-1373.8390	2.3966	3.8641	-1373.9609	-1373.8651
	aug-cc-pvdz	3.1968	3.2173	-1373.8240	-1373.6974	2.4364	3.8229	-1373.8192	-1373.7228
	aug-cc-pvtz	3.1446	3.1696	-1373.9720	-1373.8463	2.3860	3.8080	-1373.9676	-1373.8722
M06HF	cc-pvdz	3.5565	3.5746	-1373.9030	-1373.7605	2.6956	3.9792	-1373.8962	-1373.7899
	cc-pvtz	3.3817	3.4078	-1374.1493	-1374.0104	2.5333	3.9291	-1374.1433	-1374.0399
	aug-cc-pvdz	3.4484	3.4809	-1373.9405	-1373.8012	2.6215	3.8435	-1373.9341	-1373.8300
	aug-cc-pvtz	3.3306	3.3679	-1374.1690	-1374.0314	2.5010	3.8508	-1374.1631	-1374.0605

Table S6: The geometry optimization with TDDFT and UDFT for TX-DBT are singlet and triplet, respectively. ΔE_1 and ΔE_2 (in ev) are the energy difference between the first excited triplet states and the second excited triplet states obtained by TDDFT and the ground states. The ground states and excited states energy calculated by UDFT are E_s and E_t (in a.u.).

		Singlet				Triplet			
		ΔE_1	ΔE_2	E_s	E_t	ΔE_1	ΔE_2	E_s	E_t
PBE	cc-pvdz	2.0178	2.1331*	-1600.4260	-1600.3429	2.1842	2.2252	-1600.4308	-1600.3448
	cc-pvtz	2.0196	2.0936*	-1600.6563	-1600.5748	2.1353	2.2237	-1600.6612	-1600.5771
	aug-cc-pvdz	2.0128	2.0846*	-1600.4689	-1600.3875	2.1212	2.2144	-1600.4734	-1600.3896
	aug-cc-pvtz	2.0141	2.0762*	-1600.6662	-1600.5853	2.1122	2.2156	-1600.6710	-1600.5878
PBE0	cc-pvdz	2.1677	2.4956	-1600.5480	-1600.4544	2.3518	2.6709	-1600.5542	-1600.4609
	cc-pvtz	2.1709	2.5106	-1600.7646	-1600.6774	2.3303	2.6222	-1600.7709	-1600.6794
	aug-cc-pvdz	2.1948	2.5239	-1600.5841	-1600.4909	2.3358	2.6274	-1600.5900	-1600.4986
	aug-cc-pvtz	2.1771	2.5178	-1600.7723	-1600.6855	2.3216	2.6076	-1600.7785	-1600.6877
B3LYP	cc-pvdz	2.2135	2.4568	-1601.7643	-1601.6762	2.3620	2.6757	-1601.7697	-1601.6772
	cc-pvtz	2.2124	2.4804	-1602.0116	-1601.9246	2.3334	2.6893	-1602.0171	-1601.9263
	aug-cc-pvdz	2.2218	2.4852	-1601.8064	-1601.7194	2.3281	2.6861	-1601.8115	-1601.7210
	aug-cc-pvtz	2.2109	2.4873	-1602.0202	-1601.9338	2.3178	2.6736	-1602.0257	-1601.9356
CAM-B3LYP	cc-pvdz	2.2051	2.7359*	-1601.3027	-1601.2067	2.4235	2.7370	-1601.3089	-1601.2038
	cc-pvtz	2.2373	2.7130*	-1601.5544	-1601.4589	2.4323	2.6933	-1601.5607	-1601.4615

	aug-cc-pvdz	2.2628	2.7066*	-1601.3457	-1601.2497	2.4451	2.6891	-1601.3515	-1601.2526
	aug-cc-pvtz	2.2521	2.6942*	-1601.5632	-1601.4679	2.4346	2.6766	-1601.5693	-1601.4710
WB97XD	cc-pvdz	2.3194	2.7617	-1601.4558	-1601.3579	2.5255	2.8724	-1601.4622	-1601.3587
	cc-pvtz	2.3294	2.7827	-1601.6773	-1601.5804	2.5164	2.8232	-1601.6838	-1601.5822
	aug-cc-pvdz	2.3560	2.7974	-1601.4907	-1601.3937	2.5298	2.8272	-1601.4966	-1601.3952
	aug-cc-pvtz	2.3385	2.7908	-1601.6855	-1601.5892	2.5148	2.8086	-1601.6918	-1601.5909
M062X	cc-pvdz	2.3556	2.7058	-1601.4164	-1601.3176	2.5711	2.9126	-1601.4231	-1601.3190
	cc-pvtz	2.3607	2.7934	-1601.6363	-1601.5384	2.5515	2.9917	-1601.6429	-1601.5406
	aug-cc-pvdz	2.3712	2.7548	-1601.4542	-1601.3559	2.5487	2.9476	-1601.4605	-1601.3529
	aug-cc-pvtz	2.3645	2.8102	-1601.6455	-1601.5479	2.5421	3.0027	-1601.6520	-1601.5451
M06HF	cc-pvdz	2.3801	2.5183	-1601.5465	-1601.4419	2.6665	2.6771	-1601.5540	-1601.4430
	cc-pvtz	2.3275	2.6965	-1601.8604	-1601.7563	2.5864	2.8386	-1601.8677	-1601.7587
	aug-cc-pvdz	2.4079	3.4276	-1601.5964	-1601.4928	2.6432	3.4653	-1601.6034	-1601.4953
	aug-cc-pvtz	2.3368	3.3632	-1601.8855	-1601.7824	2.5799	3.3801	-1601.8927	-1601.7852

Table S7: The geometry optimization with TDDFT and UDFT for BDBT are singlet and triplet, respectively. ΔE_1 and ΔE_2 (in ev) are the energy difference between the first excited triplet states and the second excited triplet states obtained by TDDFT and the ground states. The ground states and excited states energy calculated by UDFT are E_s and E_t (in a.u.).

		Singlet				Triplet			

		ΔE_1	ΔE_2	E_s	E_t	ΔE_1	ΔE_2	E_s	E_t
PBE	cc-pvdz	1.8249	2.4254	-1203.5673	-1203.4886	1.8474	2.4524	-1203.5680	-1203.4887
	cc-pvtz	1.8512	2.3855	-1203.7864	-1203.7082	1.8718	2.4118	-1203.7872	-1203.7084
	aug-cc-pvdz	1.8571	2.3732	-1203.6090	-1203.5305	1.8767	2.3984	-1203.6096	-1203.5306
	aug-cc-pvtz	1.8592	2.3641	-1203.7960	-1203.7180	1.8787	2.3900	-1203.7968	-1203.7182
PBE0	cc-pvdz	2.0358	2.5421	-1203.6659	-1203.5843	2.0693	2.5884	-1203.6670	-1203.5845
	cc-pvtz	2.0653	2.5468	-1203.8702	-1203.7888	2.0975	2.5918	-1203.8713	-1203.7891
	aug-cc-pvdz	2.0940	2.5659	-1203.7004	-1203.6181	2.1243	2.6112	-1203.7014	-1203.6183
	aug-cc-pvtz	2.0824	2.5506	-1203.8776	-1203.7963	2.1135	2.5952	-1203.8787	-1203.7966
B3LYP	cc-pvdz	2.0799	2.5879	-1204.7428	-1204.6591	2.1083	2.6380	-1204.7437	-1204.6593
	cc-pvtz	2.1178	2.5951	-1204.9797	-1204.8959	2.1446	2.6444	-1204.9807	-1204.8961
	aug-cc-pvdz	2.1363	2.6019	-1204.7841	-1204.6999	2.1614	2.6509	-1204.7850	-1204.7000
	aug-cc-pvtz	2.1340	2.5945	-1204.9881	-1204.9043	2.1595	2.6433	-1204.9891	-1204.9045
CAM-B3LYP	cc-pvdz	2.1281	2.6667	-1204.2813	-1204.1954	2.1708	2.7036	-1204.2826	-1204.1956
	cc-pvtz	2.1735	2.7006	-1204.5222	-1204.4359	2.2148	2.7359	-1204.5234	-1204.4362
	aug-cc-pvdz	2.1989	2.7113	-1204.3235	-1204.2365	2.2385	2.7463	-1204.3247	-1204.2367
	aug-cc-pvtz	2.1934	2.7090	-1204.5307	-1204.4443	2.2337	2.7434	-1204.5319	-1204.4446
WB97XD	cc-pvdz	2.2408	2.7427	-1204.4525	-1204.3641	2.2786	2.7858	-1204.4537	-1204.3643
	cc-pvtz	2.2707	2.7541	-1204.6632	-1204.5752	2.3080	2.7952	-1204.6644	-1204.5755
	aug-cc-pvdz	2.2996	2.7730	-1204.4862	-1204.3971	2.3350	2.8143	-1204.4873	-1204.3973

	aug-cc-pvtz	2.2873	2.7596	-1204.6711	-1204.5830	2.3238	2.7999	-1204.6724	-1204.5833
M062X	cc-pvdz	2.2900	2.7933	-1204.4165	-1204.3263	2.3182	2.8503	-1204.4178	-1204.3264
	cc-pvtz	2.3543	2.8091	-1204.6226	-1204.5323	2.3850	2.8601	-1204.6239	-1204.5325
	aug-cc-pvdz	2.3455	2.8098	-1204.4533	-1204.3626	2.3722	2.8641	-1204.4545	-1204.3627
	aug-cc-pvtz	2.3709	2.8126	-1204.6312	-1204.5410	2.4012	2.8622	-1204.6325	-1204.5412
M06HF	cc-pvdz	2.1109	2.8424	-1204.5497	-1204.4309	2.1232	2.9155	-1204.5512	-1204.4288
	cc-pvtz	2.2469	2.7528	-1204.8376	-1204.7177	2.2628	2.8169	-1204.8391	-1204.7190
	aug-cc-pvdz	2.2076	2.8504	-1204.5974	-1204.5045	2.2180	2.9195	-1204.5988	-1204.5047
	aug-cc-pvtz	2.2672	2.7541	-1204.8610	-1204.7699	2.2827	2.8167	-1204.8625	-1204.7701

Table S8: The geometry optimization with TDDFT and UDFT for FBDBT are singlet and triplet, respectively. ΔE_1 and ΔE_2 (in ev) are the energy difference between the first excited triplet states and the second excited triplet states obtained by TDDFT and the ground states. The ground states and excited states energy calculated by UDFT are E_s and E_t (in a.u.).

		Singlet				Triplet			
		ΔE_1	ΔE_2	E_s	E_t	ΔE_1	ΔE_2	E_s	E_t
	cc-pvdz	1.8611	2.4468	-1302.7265	-1302.6469	1.8889	2.4773	-1302.7274	-1302.6470
PBE	cc-pvtz	1.8855	2.4045	-1302.9789	-1302.8998	1.9115	2.4338	-1302.9799	-1302.9000
	aug-cc-pvdz	1.8921	2.3886	-1302.7779	-1302.6985	1.9171	2.4165	-1302.7787	-1302.6986

	aug-cc-pvtz	1.8954	2.3806	-1302.9909	-1302.9120	1.9203	2.4092	-1302.9919	-1302.9122
PBE0	cc-pvdz	2.0593	2.5655	-1302.8203	-1302.7376	2.0968	2.6142	-1302.8216	-1302.7378
	cc-pvtz	2.0888	2.5718	-1303.0572	-1302.9748	2.1252	2.6191	-1303.0586	-1302.9751
	aug-cc-pvdz	2.1165	2.5893	-1302.8636	-1302.7803	2.1511	2.6364	-1302.8648	-1302.7806
	aug-cc-pvtz	2.1054	2.5756	-1303.0667	-1302.9842	2.1408	2.6222	-1303.0680	-1302.9845
B3LYP	cc-pvdz	2.1032	2.6037	-1303.9849	-1303.9003	2.1361	2.6554	-1303.9860	-1303.9004
	cc-pvtz	2.1408	2.6118	-1304.2553	-1304.1706	2.1723	2.6623	-1304.2564	-1304.1708
	aug-cc-pvdz	2.1587	2.6175	-1304.0353	-1303.9502	2.1886	2.6674	-1304.0364	-1303.9503
	aug-cc-pvtz	2.1565	2.6113	-1304.2658	-1304.1811	2.1869	2.6610	-1304.2670	-1304.1814
CAM-B3LYP	cc-pvdz	2.1438	2.6927	-1303.5060	-1303.4190	2.1899	2.7331	-1303.5074	-1303.4192
	cc-pvtz	2.1878	2.7271	-1303.7811	-1303.6937	2.2326	2.7659	-1303.7825	-1303.6941
	aug-cc-pvdz	2.2117	2.7368	-1303.5578	-1303.4698	2.2548	2.7750	-1303.5592	-1303.4701
	aug-cc-pvtz	2.2065	2.7352	-1303.7918	-1303.7044	2.2503	2.7731	-1303.7933	-1303.7047
WB97XD	cc-pvdz	2.2575	2.7646	-1303.6688	-1303.5794	2.2991	2.8102	-1303.6702	-1303.5796
	cc-pvtz	2.2872	2.7778	-1303.9126	-1303.8235	2.3284	2.8214	-1303.9141	-1303.8239
	aug-cc-pvdz	2.3146	2.7951	-1303.7108	-1303.6206	2.3542	2.8385	-1303.7121	-1303.6208
	aug-cc-pvtz	2.3031	2.7833	-1303.9225	-1303.8333	2.3436	2.8260	-1303.9240	-1303.8337
M062X	cc-pvdz	2.3106	2.8009	-1303.6341	-1303.5427	2.3436	2.8585	-1303.6354	-1303.5429
	cc-pvtz	2.3725	2.8243	-1303.8734	-1303.7820	2.4083	2.8755	-1303.8748	-1303.7822
	aug-cc-pvdz	2.3668	2.8212	-1303.6790	-1303.5871	2.3988	2.8756	-1303.6803	-1303.5872

	aug-cc-pvtz	2.3888	2.8290	-1303.8839	-1303.7925	2.4244	2.8787	-1303.8853	-1303.7928
M06HF	cc-pvdz	2.1352	2.8267	-1303.7666	-1303.6447	2.1506	2.9016	-1303.7683	-1303.6454
	cc-pvtz	2.2687	2.7496	-1304.0982	-1303.9791	2.2891	2.8139	-1304.0998	-1303.9783
	aug-cc-pvdz	2.2334	2.8393	-1303.8237	-1303.7299	2.2472	2.9097	-1303.8253	-1303.7301
	aug-cc-pvtz	2.2904	2.7537	-1304.1237	-1304.0316	2.3105	2.8162	-1304.1253	-1304.0318

Table S9: The geometry optimization with TDDFT and UDFT for ClBDBT are singlet and triplet, respectively. ΔE_1 and ΔE_2 (in ev) are the energy difference between the first excited triplet states and the second excited triplet states obtained by TDDFT and the ground states. The ground states and excited states energy calculated by UDFT are E_s and E_t (in a.u.).

		Singlet				Triplet			
		ΔE_1	ΔE_2	E_s	E_t	ΔE_1	ΔE_2	E_s	E_t
PBE	cc-pvdz	1.8114	2.3820	-1662.9933	-1662.9154	1.8300	2.4075	-1662.9940	-1662.9155
	cc-pvtz	1.8345	2.3439	-1663.2271	-1663.1496	1.8507	2.3688	-1663.2279	-1663.1498
	aug-cc-pvdz	1.8429	2.3314	-1663.0378	-1662.9600	1.8579	2.3560	-1663.0384	-1662.9601
	aug-cc-pvtz	1.8434	2.3239	-1663.2372	-1663.1599	1.8583	2.3493	-1663.2379	-1663.1601
PBE0	cc-pvdz	2.0210	2.5312	-1663.1252	-1663.0439	2.0551	2.5758	-1663.1262	-1663.0440
	cc-pvtz	2.0499	2.5378	-1663.3439	-1663.2629	2.0827	2.5802	-1663.3450	-1663.2632
	aug-cc-pvdz	2.0790	2.5580	-1663.1621	-1663.0802	2.1094	2.6005	-1663.1631	-1663.0804
	aug-cc-pvtz	2.0671	2.5429	-1663.3517	-1663.2707	2.0986	2.5844	-1663.3528	-1663.2710

B3LYP	cc-pvdz	2.0645	2.5726	-1664.3604	-1664.2773	2.0925	2.6226	-1664.3613	-1664.2774
	cc-pvtz	2.1010	2.5821	-1664.6114	-1664.5282	2.1270	2.6310	-1664.6124	-1664.5284
	aug-cc-pvdz	2.1203	2.5905	-1664.4042	-1664.3204	2.1444	2.6393	-1664.4050	-1664.3206
	aug-cc-pvtz	2.1174	2.5834	-1664.6202	-1664.5369	2.1419	2.6319	-1664.6212	-1664.5372
CAM-B3LYP	cc-pvdz	2.1121	2.6594	-1663.9017	-1663.8160	2.1576	2.6901	-1663.9029	-1663.8162
	cc-pvtz	2.1563	2.6939	-1664.1571	-1664.0711	2.2001	2.7194	-1664.1584	-1664.0714
	aug-cc-pvdz	2.1821	2.7055	-1663.9465	-1663.8597	2.2239	2.7295	-1663.9476	-1663.8599
	aug-cc-pvtz	2.1762	2.7028	-1664.1660	-1664.0799	2.2188	2.7254	-1664.1673	-1664.0802
WB97XD	cc-pvdz	2.2255	2.7322	-1664.0550	-1663.9670	2.2655	2.7709	-1664.0562	-1663.9672
	cc-pvtz	2.2552	2.7454	-1664.2802	-1664.1925	2.2947	2.7802	-1664.2815	-1664.1928
	aug-cc-pvdz	2.2840	2.7647	-1664.0910	-1664.0022	2.3214	2.7991	-1664.0922	-1664.0024
	aug-cc-pvtz	2.2718	2.7516	-1664.2886	-1664.2007	2.3105	2.7848	-1664.2898	-1664.2011
M062X	cc-pvdz	2.2801	2.7766	-1664.0088	-1663.9187	2.3094	2.8347	-1664.0100	-1663.9188
	cc-pvtz	2.3417	2.7975	-1664.2328	-1664.1427	2.3739	2.8478	-1664.2340	-1664.1428
	aug-cc-pvdz	2.3358	2.7969	-1664.0478	-1663.9572	2.3635	2.8514	-1664.0490	-1663.9573
	aug-cc-pvtz	2.3583	2.8025	-1664.2420	-1664.1519	2.3901	2.8511	-1664.2432	-1664.1521
M06HF	cc-pvdz	2.1059	2.8202	-1664.1378	-1664.0183	2.1172	2.8989	-1664.1393	-1664.0463
	cc-pvtz	2.2402	2.7337	-1664.4585	-1664.3674	2.2561	2.8010	-1664.4600	-1664.3676
	aug-cc-pvdz	2.2032	2.8307	-1664.1887	-1664.0961	2.2124	2.9044	-1664.1902	-1664.0962
	aug-cc-pvtz	2.2617	2.7369	-1664.4837	-1664.3928	2.2774	2.8021	-1664.4852	-1664.3929

Table S10: The geometry optimization with TDDFT and UDFT for BrBDBT are singlet and triplet, respectively. ΔE_1 and ΔE_2 (in ev) are the energy difference between the first excited triplet states and the second excited triplet states obtained by TDDFT and the ground states. The ground states and excited states energy calculated by UDFT are E_s and E_t (in a.u.).

		Singlet				Triplet			
		ΔE_1	ΔE_2	E_s	E_t	ΔE_1	ΔE_2	E_s	E_t
PBE	cc-pvdz	1.8058	2.3747	-3776.7176	-3776.6400	1.8249	2.4001	-3776.7183	-3776.6401
	cc-pvtz	1.8290	2.3341	-3777.0087	-3776.9315	1.8444	2.3596	-3777.0094	-3776.9317
	aug-cc-pvdz	1.8366	2.3230	-3776.7621	-3776.6846	1.8521	2.3473	-3776.7627	-3776.6847
	aug-cc-pvtz	1.8392	2.3167	-3777.0186	-3776.9416	1.8545	2.3419	-3777.0194	-3776.9418
PBE0	cc-pvdz	2.0157	2.5246	-3776.9005	-3776.8194	2.0506	2.5694	-3776.9016	-3776.8196
	cc-pvtz	2.0440	2.5307	-3777.1772	-3777.0965	2.0774	2.5732	-3777.1784	-3777.0968
	aug-cc-pvdz	2.0733	2.5510	-3776.9375	-3776.8558	2.1042	2.5935	-3776.9385	-3776.8560
	aug-cc-pvtz	2.0618	2.5363	-3777.1849	-3777.1041	2.0939	2.5778	-3777.1861	-3777.1044
B3LYP	cc-pvdz	2.0597	2.5665	-3778.2961	-3778.2132	2.0884	2.6164	-3778.2970	-3778.2133
	cc-pvtz	2.0954	2.5754	-3778.6052	-3778.5222	2.1219	2.6243	-3778.6062	-3778.5224
	aug-cc-pvdz	2.1149	2.5841	-3778.3399	-3778.2564	2.1395	2.6327	-3778.3408	-3778.2566
	aug-cc-pvtz	2.1124	2.5775	-3778.6138	-3778.5307	2.1376	2.6258	-3778.6148	-3778.5310
CAM-B3LYP	cc-pvdz	2.1087	2.6541	-3777.9355	-3777.8500	2.1549	2.6855	-3777.9367	-3777.8502
	cc-pvtz	2.1525	2.6880	-3778.2490	-3778.1632	2.1970	2.7143	-3778.2503	-3778.1635

	aug-cc-pvdz	2.1785	2.6997	-3777.9803	-3777.8937	2.2209	2.7244	-3777.9814	-3777.8939
	aug-cc-pvtz	2.1730	2.6974	-3778.2577	-3778.1718	2.2162	2.7208	-3778.2590	-3778.1721
WB97XD	cc-pvdz	2.2217	2.7267	-3778.0387	-3777.9508	2.2625	2.7661	-3778.0399	-3777.9510
	cc-pvtz	2.2511	2.7394	-3778.3227	-3778.2352	2.2912	2.7749	-3778.3240	-3778.2355
	aug-cc-pvdz	2.2799	2.7587	-3778.0749	-3777.9862	2.3179	2.7937	-3778.0760	-3777.9865
	aug-cc-pvtz	2.2681	2.7459	-3778.3310	-3778.2433	2.3073	2.7797	-3778.3322	-3778.2436
M062X	cc-pvdz	2.2747	2.7709	-3777.9960	-3777.9061	2.3046	2.8299	-3777.9972	-3777.9062
	cc-pvtz	2.3368	2.7919	-3778.2662	-3778.1763	2.3697	2.8429	-3778.2675	-3778.1765
	aug-cc-pvdz	2.3303	2.7910	-3778.0355	-3777.9451	2.3585	2.8462	-3778.0367	-3777.9452
	aug-cc-pvtz	2.3538	2.7970	-3778.2753	-3778.1855	2.3862	2.8463	-3778.2766	-3778.1857
M06HF	cc-pvdz	2.0991	2.8177	-3778.3269	-3778.2039	2.1107	2.8979	-3778.3284	-3778.2073
	cc-pvtz	2.2354	2.7319	-3778.6720	-3778.5540	2.2515	2.8009	-3778.6735	-3778.5528
	aug-cc-pvdz	2.1964	2.8283	-3778.3785	-3778.2862	2.2058	2.9035	-3778.3800	-3778.2863
	aug-cc-pvtz	2.2572	2.7353	-3778.6970	-3778.6063	2.2730	2.8021	-3778.6985	-3778.6065

Table S11: The geometry optimization with TDDFT and UDFT for XA are singlet and triplet, respectively. ΔE_1 and ΔE_2 (in ev) are the energy difference between the first excited triplet states and the second excited triplet states obtained by TDDFT and the ground states. The ground states and excited states energy calculated by UDFT are E_s and E_t (in a.u.).

		Singlet	Triplet

		ΔE_1	ΔE_2	E_s	E_t	ΔE_1	ΔE_2	E_s	E_t
PBE	cc-pvdz	1.9657	2.4873*	-649.9213	-649.8013	1.9723	2.4887*	-649.9215	-649.7000
	cc-pvtz	1.9880	2.4771*	-650.0894	-649.9950	1.9940	2.4781*	-650.0895	-649.9950
	aug-cc-pvdz	1.9836	2.4791*	-649.9553	-649.2310	1.9896	2.4806*	-649.9554	-649.8305
	aug-cc-pvtz	1.9876	2.4743*	-650.0973	-649.8595	1.9986	2.4757*	-650.0973	-650.0031
PBE0	cc-pvdz	2.3801	2.4664	-649.9600	-649.8665	2.3769	2.4663	-649.9598	-649.8665
	cc-pvtz	2.3975	2.4908	-650.1175	-650.0251	2.3947	2.4905	-650.1172	-650.0249
	aug-cc-pvdz	2.4320	2.5045	-649.9888	-649.8955	2.4300	2.5044	-649.9886	-649.8953
	aug-cc-pvtz	2.4139	2.5016	-650.1238	-650.0316	2.4115	2.5012	-650.1235	-650.0314
B3LYP	cc-pvdz	2.4312	2.4545*	-650.6943	-650.5972	2.4319	2.4528*	-650.6943	-650.5973
	cc-pvtz	2.4656	2.4805*	-650.8754	-650.7786	2.4660	2.4792*	-650.8752	-650.7785
	aug-cc-pvdz	2.4715	2.4997*	-650.7280	-650.6308	2.4721	2.4990*	-650.7279	-650.6307
	aug-cc-pvtz	2.4767	2.4934*	-650.8825	-650.7834	2.4771	2.4925*	-650.8823	-650.7831
CAM-B3LYP	cc-pvdz	2.4071	2.7283	-650.3558	-650.2503	2.4035	2.7242	-650.3557	-650.2502
	cc-pvtz	2.4547	2.7760	-650.5400	-650.4344	2.4519	2.7717	-650.5397	-650.4341
	aug-cc-pvdz	2.4824	2.7854	-650.3904	-650.2846	2.4803	2.7815	-650.3902	-650.2843
	aug-cc-pvtz	2.4764	2.7903	-650.5472	-650.4415	2.4740	2.7860	-650.5468	-650.4412
WB97XD	cc-pvdz	2.5382	2.7565	-650.4739	-650.3657	2.5354	2.7530	-650.4737	-650.3655
	cc-pvtz	2.5568	2.7862	-650.6366	-650.5289	2.5546	2.7826	-650.6363	-650.5286
	aug-cc-pvdz	2.5897	2.8023	-650.5017	-650.3935	2.5881	2.7989	-650.5015	-650.3932

	aug-cc-pvtz	2.5718	2.7977	-650.6432	-650.5355	2.5699	2.7941	-650.6429	-650.5352
M062X	cc-pvdz	2.5987	2.6981	-650.4607	-650.3504	2.5952	2.6919	-650.4606	-650.3502
	cc-pvtz	2.6143	2.7918	-650.6174	-650.5184	2.6112	2.7856	-650.6172	-650.5182
	aug-cc-pvdz	2.6311	2.7504	-650.4905	-650.3913	2.6290	2.7445	-650.4903	-650.3912
	aug-cc-pvtz	2.6257	2.8100	-650.6242	-650.5256	2.6230	2.8037	-650.6239	-650.5255
M06HF	cc-pvdz	2.5058	2.6206*	-650.5541	-650.4383	2.4936	2.6152*	-650.5539	-650.4382
	cc-pvtz	2.5757	2.6767	-650.7653	-650.6488	2.5702	2.6648	-650.7650	-650.6486
	aug-cc-pvdz	2.6610	3.8384	-650.5925	-650.4760	2.6036	2.6579*	-650.5923	-650.4757
	aug-cc-pvtz	2.5914	3.8096	-650.7823	-650.6660	2.5865	2.6932	-650.7820	-650.6658

Table S12: The geometry optimization with TDDFT and UDFT for AR are singlet and triplet, respectively. ΔE_1 and ΔE_2 (in ev) are the energy difference between the first excited triplet states and the second excited triplet states obtained by TDDFT and the ground states. The ground states and excited states energy calculated by UDFT are E_s and E_t (in a.u.).

		Singlet				Triplet			
		ΔE_1	ΔE_2	E_s	E_t	ΔE_1	ΔE_2	E_s	E_t
PBE	cc-pvdz	2.0977	2.2270*	-630.0823	-629.9137	1.9189	2.1920*	-630.0773	-629.9287
	cc-pvtz	2.1163	2.2074*	-630.2435	-630.0655	1.9397	2.1799*	-630.2388	-630.0908
	aug-cc-pvdz	2.1111	2.2012*	-630.1162	-629.9114	1.9363	2.1788*	-630.1114	-629.9551
	aug-cc-pvtz	2.1197	2.2000*	-630.2515	-630.0604	1.9453	2.1769*	-630.2469	-630.0837

PBE0	cc-pvdz	2.2913	2.6618	-630.1273	-630.0366	2.1862	2.4651	-630.1220	-630.0286
	cc-pvtz	2.2882	2.6834	-630.2773	-630.1875	2.2010	2.4917	-630.2723	-630.1799
	aug-cc-pvdz	2.2968	2.6964	-630.1555	-630.0658	2.2247	2.5086	-630.1505	-630.0571
	aug-cc-pvtz	2.2900	2.6929	-630.2837	-630.1942	2.2141	2.5050	-630.2788	-630.1866
B3LYP	cc-pvdz	2.3106	2.6114	-630.8444	-630.7542	2.2253	2.4174	-630.8393	-630.7424
	cc-pvtz	2.3106	2.6432	-631.0189	-630.9293	2.2428	2.4531	-631.0141	-630.9262
	aug-cc-pvdz	2.3103	2.6483	-630.8783	-630.7891	2.2543	2.4622	-630.8735	-630.7856
	aug-cc-pvtz	2.3093	2.6530	-631.0260	-630.9368	2.2516	2.4668	-631.0214	-630.9336
CAM-B3LYP	cc-pvdz	2.3756	2.9432	-630.5029	-630.4053	2.2505	2.7456	-630.4978	-630.4029
	cc-pvtz	2.3929	2.9876	-630.6803	-630.5834	2.2920	2.7956	-630.6756	-630.5809
	aug-cc-pvdz	2.3997	2.9959	-630.5375	-630.4410	2.3140	2.8092	-630.5328	-630.4382
	aug-cc-pvtz	2.3987	2.9998	-630.6875	-630.5910	2.3111	2.8128	-630.6829	-630.5884
WB97XD	cc-pvdz	2.4558	2.9704	-630.6318	-630.5321	2.3468	2.7712	-630.6266	-630.5294
	cc-pvtz	2.4547	2.9970	-630.7869	-630.6881	2.3639	2.8039	-630.7820	-630.6854
	aug-cc-pvdz	2.4633	3.0122	-630.6594	-630.5608	2.3864	2.8231	-630.6544	-630.5577
	aug-cc-pvtz	2.4567	3.0068	-630.7935	-630.6951	2.3764	2.8181	-630.7887	-630.6923
M062X	cc-pvdz	2.4435	2.9220	-630.6139	-630.5131	2.3306	2.7285	-630.6084	-630.5101
	cc-pvtz	2.4511	3.0113	-630.7634	-630.6631	2.3562	2.8246	-630.7583	-630.6601
	aug-cc-pvdz	2.4380	2.9709	-630.6438	-630.5445	2.3551	2.7886	-630.6386	-630.5411
	aug-cc-pvtz	2.4509	3.0279	-630.7704	-630.6707	2.3666	2.8464	-630.7654	-630.6672

M06HF	cc-pvdz	2.4449	2.7767	-630.7105	-630.6015	2.2973	2.6168	-630.7047	-630.6000
	cc-pvtz	2.4314	2.9415	-630.9146	-630.8062	2.3035	2.7951	-630.9095	-630.8040
	aug-cc-pvdz	2.4453	2.8844	-630.7497	-630.6421	2.3425	3.6765	-630.7443	-630.6394
	aug-cc-pvtz	2.4362	3.6954	-630.9322	-630.8247	2.3215	3.6173	-630.9271	-630.8221

Table S13: The geometry optimizationwith TDDFT and UDFT for AD are singlet and triplet, respectively. ΔE_1 and ΔE_2 (in ev) are the energy difference between the first excited triplet states and the second excited triplet states obtained by TDDFT and the ground states. The ground states and excited states energy calculated by UDFT are E_s and E_t (in a.u.).

		Singlet				Triplet			
		ΔE_1	ΔE_2	E_s	E_t	ΔE_1	ΔE_2	E_s	E_t
PBE	cc-pvdz	1.8982	2.1355*	-972.7888	-972.6461	2.1524	2.2542*	-972.7958	-972.6418
	cc-pvtz	1.9086	2.1142*	-972.9504	-972.8097	2.1568	2.2202*	-972.9575	-972.7947
	aug-cc-pvdz	1.9010	2.1165*	-972.8206	-972.6729	2.1483	2.2139*	-972.8272	-972.6133
	aug-cc-pvtz	1.9034	2.1061*	-972.9576	-972.8114	2.1508	2.2046*	-972.9646	-972.7986
PBE0	cc-pvdz	2.0385	2.3643	-972.8600	-972.7745	2.2991	2.6334	-972.8689	-972.7763
	cc-pvtz	2.0557	2.3855	-973.0112	-972.9264	2.2913	2.6468	-973.0201	-972.9288
	aug-cc-pvdz	2.0907	2.4004	-972.8867	-972.8015	2.3071	2.6560	-972.8951	-972.8037
	aug-cc-pvtz	2.0704	2.3952	-973.0169	-972.9323	2.2909	2.6511	-973.0257	-972.9349
B3LYP	cc-pvdz	2.1099	2.3264	-973.6697	-973.5839	2.3350	2.5955	-973.6776	-973.5853

	cc-pvtz	2.1273	2.3555	-973.8437	-973.7585	2.3255	2.6183	-973.8517	-973.7606
	aug-cc-pvdz	2.1476	2.3619	-973.7012	-973.6158	2.3297	2.6186	-973.7087	-973.6177
	aug-cc-pvtz	2.1361	2.3649	-973.8501	-973.7652	2.3194	2.6222	-973.8580	-973.7674
CAM-B3LYP	cc-pvdz	2.0552	2.6083	-973.3433	-973.2521	2.3487	2.8717	-973.3523	-973.2530
	cc-pvtz	2.1030	2.6518	-973.5204	-973.4296	2.3654	2.9065	-973.5294	-973.4314
	aug-cc-pvdz	2.1361	2.6633	-973.3756	-973.2845	2.3809	2.9100	-973.3841	-973.2862
	aug-cc-pvtz	2.1253	2.6657	-973.5268	-973.4363	2.3710	2.9132	-973.5357	-973.4384
WB97XD	cc-pvdz	2.1783	2.6353	-973.4538	-973.3600	2.4540	2.8998	-973.4629	-973.3613
	cc-pvtz	2.1999	2.6627	-973.6090	-973.5160	2.4512	2.9183	-973.6182	-973.5181
	aug-cc-pvdz	2.2342	2.6801	-973.4796	-973.3861	2.4685	2.9292	-973.4883	-973.3881
	aug-cc-pvtz	2.2151	2.6743	-973.6150	-973.5221	2.4526	2.9236	-973.6241	-973.5245
M062X	cc-pvdz	2.2079	2.5807	-973.4251	-973.3302	2.5014	2.8360	-973.4347	-973.3320
	cc-pvtz	2.2273	2.6740	-973.5789	-973.4843	2.4913	2.9191	-973.5883	-973.4869
	aug-cc-pvdz	2.2455	2.6374	-973.4531	-973.3587	2.4943	2.8756	-973.4622	-973.3611
	aug-cc-pvtz	2.2385	2.6939	-973.5854	-973.4911	2.4868	2.9318	-973.5947	-973.4940
M06HF	cc-pvdz	2.1825	2.4182	-973.5119	-973.4131	2.5626	2.6119	-973.5226	-973.4142
	cc-pvtz	2.1452	2.6033	-973.7291	-973.6297	2.4872	2.7765	-973.7394	-973.6323
	aug-cc-pvdz	2.2384	3.6148	-973.5485	-973.4495	2.5511	3.7475	-973.5586	-973.4522
	aug-cc-pvtz	2.1610	3.5809	-973.7464	-973.6477	2.4829	3.7016	-973.7566	-973.6509

Table S14: The energy differences ΔE (in eV) relative to experimental values for the phosphorescence energy of AQs (except for BDBT, FBDBT, BrBDBT) calculated by TD//TDDFT with different functionals (PBE, PBE0, B3LYP, CAM-B3LYP, ωB97XD, M062X, M06HF) at the cc-pVTZ level.

	Expt.	CC2	PBE	PBE0	B3LYP	CAM-B3LYP	wB97XD	M062X	M06HF
HA	2.53	0.01	-0.86*	-0.79	-0.73	-0.67	-0.56	-0.40	-0.23*
AAT	1.94	0.09	-0.69	-0.59	-0.57	-0.44	-0.37	-0.24	0.01
BP	2.58	-0.12	-0.74	-0.51	-0.46	-0.40	-0.30	-0.23	-0.36
TX-BT	2.43	0.89	0.27	0.23	0.33	0.19	0.35	0.73	0.95
TX-DBT	2.34	0.48	-0.25*	-0.17	-0.13	0.37	-0.01	0.02	-0.01
BDBT	2.18	0.32	-0.32	-0.11	-0.06	0.00	0.10	0.18	0.07
FBDBT	2.18	0.34	-0.29	-0.09	-0.03	0.01	0.11	0.20	0.09
ClBDBT	2.64	-0.15	-0.80	-0.59	-0.54	-0.48	-0.38	-0.30	-0.40
BrBDBT	2.18	0.30	-0.35	-0.13	-0.08	-0.01	0.08	0.16	0.03
XA	3.10	0.06	-0.62*	-0.70	-0.62	-0.65	-0.54	-0.49	-0.52
AR	2.36	0.50	-0.15*	-0.07	-0.05	0.03	0.09	0.09	0.07
AD	2.73	0.06	-0.62*	-0.68	-0.60	-0.63	-0.53	-0.50	-0.59
MUE	-	0.23	0.50	0.39	0.35	0.32	0.29	0.29	0.28

Table S15: The energy differences ΔE (in eV) relative to experimental values for the phosphorescence energy of AQs (except for BDBT, FBDBT, BrBDBT) calculated by TD//UDFT with different functionals (PBE, PBE0, B3LYP, CAM-B3LYP, ωB97XD, M062X, M06HF) at the cc-pVTZ level.

	PBE	PBE0	B3LYP	CAM-B3LYP	wB97XD	M062X	M06HF
HA	1.18	0.52	0.46	0.35	0.30	0.21	0.08
AAT	0.32	0.33	0.29	0.26	0.22	0.15	0.09
BP	0.34	0.39	0.30	0.27	0.22	0.16	0.14
TX-BT	0.44	0.63	0.62	0.78	0.85	1.01	1.35
TX-DBT	0.12	0.03	0.03	0.26	0.30	0.32	0.49
BDBT	0.05	0.04	0.10	0.17	0.22	0.28	1.09

FBDBT	0.02	0.07	0.13	0.20	0.25	0.31	1.06
ClBDBT	0.53	0.43	0.37	0.30	0.25	0.19	0.16
BrBDBT	0.08	0.02	0.08	0.16	0.21	0.27	1.04
XA	0.53	0.58	0.47	0.23	0.17	0.40	0.07
AR	2.48	0.08	0.07	0.27	0.33	0.37	0.59
AD	1.09	0.43	0.41	0.26	0.20	0.16	0.03
MUE	0.60	0.30	0.28	0.29	0.29	0.32	0.52

Table S16: The energy differences ΔE (in eV) relative to experimental values for the phosphorescence energy of AQs (except for BDBT, FBDBT, BrBDBT) calculated by U//UDFT with different functionals (PBE, PBE0, B3LYP, CAM-B3LYP, ωB97XD, M062X, M06HF) at the cc-pVTZ level.

	CC2	PBE	PBE0	B3LYP	CAM-B3LYP	wB97XD	M062X	M06HF
HA	0.01	1.48	-0.47	-0.46	-0.35	-0.30	-0.21	-0.08
AAT	0.09	-0.31	-0.32	-0.28	-0.25	-0.21	-0.14	-0.08
BP	-0.08	-0.32	-0.36	-0.27	-0.24	-0.19	-0.13	-0.10
TX-BT	0.24	-0.11	-0.10	-0.08	-0.06	0.01	0.18	0.38
TX-DBT	0.57	-0.05	0.15	0.13	0.36	0.42	0.45	0.62
BDBT	0.34	-0.03	0.06	0.13	0.20	0.25	0.31	1.09
FBDBT	0.39	0.00	0.10	0.15	0.23	0.28	0.35	1.13
ClBDBT	-0.12	-0.52	-0.41	-0.35	-0.27	-0.23	-0.16	-0.12
BrBDBT	0.33	-0.06	0.04	0.10	0.19	0.23	0.30	1.11
XA	0.06	-0.53	-0.59	-0.47	-0.22	-0.17	-0.41	0.07
AR	0.47	1.66	0.15	0.03	0.21	0.27	0.31	0.51
AD	0.20	1.70	-0.25	-0.25	-0.07	-0.01	0.03	0.18
MUE	0.21	0.56	0.25	0.23	0.22	0.21	0.25	0.46

Table S17: The energy differences ΔE (in eV) relative to experimental values for the phosphorescence energy of AQs (except for BDBT, FBDBT, BrBDBT) calculated by TD//UDFT with different functionals (PBE, PBE0, B3LYP, CAM-B3LYP, ωB97XD, M062X, M06HF) at the cc-pVTZ level.

	PBE	PBE0	B3LYP	CAM-	wB97XD	M062X	M06HF

	B3LYP						
HA	-0.86*	-0.78	-0.73	-0.66	-0.56	-0.40	-0.23*
AAT	-0.66	-0.58	-0.55	-0.44	-0.36	-0.24	-0.02
BP	-0.71	-0.47	-0.42	-0.35	-0.26	-0.20	-0.35
TX-BT	-0.25	-0.47	-0.35	-0.59	-0.44	-0.03	0.10
TX-DBT	-0.20	-0.01	-0.01	0.09	0.18	0.21	0.25
BDBT	-0.30	-0.08	-0.03	0.04	0.13	0.21	0.09
FBDBT	-0.26	-0.05	0.00	0.06	0.15	0.23	0.11
ClBDBT	-0.79	-0.56	-0.51	-0.44	-0.34	-0.26	-0.38
BrBDBT	-0.33	-0.10	-0.05	0.02	0.12	0.19	0.08
XA	-0.62*	-0.71	-0.62*	-0.65	-0.55	-0.49	-0.53
AR	-0.18*	-0.16	-0.12	-0.07	0.00	-0.01	-0.06
AD	-0.51*	-0.44	-0.41	-0.37	-0.28	-0.24	-0.24
MUE	0.47	0.37	0.32	0.31	0.28	0.23	0.20

Table S18: The energy differences ΔE (in eV) relative to CC2 for the phosphorescence energy of AQs calculated by U//TDDFT with different functionals (PBE, PBE0, B3LYP, CAM-B3LYP, ωB97XD, M062X, M06HF) at the cc-pVTZ level.

	PBE	PBE0	B3LYP	CAM-B3LYP	wB97XD	M062X	M06HF
HA	1.17	-0.53	-0.47	-0.36	-0.31	-0.23	-0.09
AAT	-0.40	-0.42	-0.38	-0.35	-0.31	-0.24	-0.18
BP	-0.23	-0.27	-0.18	-0.15	-0.11	-0.05	-0.03
TX-BT	-0.45	-0.27	-0.27	-0.11	-0.05	0.11	0.46
TX-DBT	-0.60	-0.45	-0.46	-0.22	-0.18	-0.16	0.01
BDBT	-0.36	-0.28	-0.21	-0.14	-0.10	-0.04	0.77
FBDBT	-0.37	-0.28	-0.21	-0.14	-0.09	-0.03	0.72
ClBDBT	-0.38	-0.29	-0.23	-0.15	-0.10	-0.04	-0.01
BrBDBT	-0.38	-0.28	-0.22	-0.15	-0.10	-0.03	0.73
XA	-0.59	-0.64	-0.52	-0.28	-0.23	-0.46	0.01
AR	1.98	-0.42	-0.42	-0.22	-0.17	-0.13	0.09
AD	1.03	-0.49	-0.47	-0.32	-0.26	-0.22	-0.09

MUE(a)	0.66	0.38	0.34	0.22	0.17	0.14	0.27

Table S19: The energy differences ΔE (in eV) relative to CC2 for the phosphorescence energy of AQs calculated by TD//TDDFT with different functionals (PBE, PBE0, B3LYP, CAM-B3LYP, ωB97XD, M062X, M06HF) at the cc-pVTZ level.

	PBE	PBE0	B3LYP	CAM-B3LYP	wB97XD	M062X	M06HF
HA	-0.87*	-0.80	-0.75	-0.68	-0.57	-0.41	-0.24*
AAT	-0.81	-0.72	-0.70	-0.56	-0.49	-0.36	-0.11
BP	-0.62	-0.40	-0.34	-0.28	-0.19	-0.12	-0.25
TX-BT	-0.62	-0.67	-0.56	-0.70	-0.55	-0.16	0.06
TX-DBT	-0.73	-0.65	-0.61	-0.11	-0.49	-0.46	-0.49
BDBT	-0.64	-0.43	-0.37	-0.32	-0.22	-0.14	-0.25
FBDBT	-0.63	-0.43	-0.38	-0.33	-0.23	-0.15	-0.25
ClBDBT	-0.66	-0.44	-0.39	-0.33	-0.23	-0.15	-0.25
BrBDBT	-0.65	-0.44	-0.38	-0.33	-0.23	-0.14	-0.24
XA	-0.68*	-0.76	-0.68*	-0.70	-0.60	-0.54	-0.58
AR	-0.65*	-0.57	-0.55	-0.47	-0.40	-0.41	-0.43
AD	-0.68*	-0.74	-0.66	-0.69	-0.59	-0.56	-0.65
MUE(a)	0.69	0.59	0.53	0.46	0.40	0.30	0.32

Table S20: The energy differences ΔE (in eV) relative to CC2 for the phosphorescence energy of AQs (except for BDBT, FBDBT, BrBDBT) calculated by U//UDFT with different functionals (PBE, PBE0, B3LYP, CAM-B3LYP, ωB97XD, M062X, M06HF) at the cc-pVTZ level.

	HA	AAT	BP	TX-BT	TX-DBT	ClBDBT	XA	AR	AD	MUE(a)
PBE	1.47	-0.40	-0.24	-0.35	-0.62	-0.39	-0.59	1.19	1.50	0.75
PBE0	-0.49	-0.41	-0.28	-0.34	-0.42	-0.29	-0.65	-0.32	-0.45	0.41
B3LYP	-0.47	-0.38	-0.18	-0.32	-0.44	-0.23	-0.53	-0.44	-0.45	0.38
CAM-B3LYP	-0.36	-0.34	-0.15	-0.30	-0.21	-0.15	-0.28	-0.26	-0.27	0.26
wB97XD	-0.31	-0.30	-0.11	-0.23	-0.14	-0.10	-0.23	-0.2	-0.20	0.20

M062X	-0.23	-0.24	-0.05	-0.07	-0.12	-0.03	-0.47	-0.16	-0.17	0.17
M06HF	-0.09	-0.17	-0.02	0.14	0.06	0.00	0.01	0.04	-0.02	0.06

Table S21: In M06HF functional the BDBT, FBDBT, ClBDBT and BrBDBT, the phosphorescence energy calculated by cc-pVDZ, cc-pVTZ, aug-cc-pVDZ, aug-cc-pVTZ and CC2.

	cc-pVDZ	cc-pVTZ	aug-cc-pVDZ	aug-cc-pVTZ	CC2
BDBT	3.33	3.27	2.56	2.51	2.52
FBDBT	3.34	3.31	2.59	2.54	2.56
ClBDBT	2.53	2.52	2.56	2.51	2.52
BrBDBT	3.29	3.29	2.55	2.50	2.51

Table S22: The S^2 values for the triplet states of AQs calculated by UDFT with different functionals (PBE, PBE0, B3LYP, CAM-B3LYP, ωB97XD, M062X, M06HF) at the cc-pVTZ level.

	PBE	PBE0	B3LYP	CAM-B3LYP	wB97XD	M062X	M06HF
HA	2.01	2.03	2.02	2.05	2.04	2.05	2.09
AAT	2.01	2.03	2.02	2.05	2.04	2.04	2.08
BP	2.01	2.04	2.02	2.05	2.04	2.02	2.03
TX-BT	2.01	2.03	2.02	2.04	2.03	2.02	2.03
TX-DBT	2.00	2.02	2.01	2.02	2.02	2.02	2.03
BDBT	2.01	2.03	2.02	2.05	2.05	2.03	2.04
FBDBT	2.01	2.03	2.02	2.06	2.05	2.03	2.04
ClBDBT	2.01	2.04	2.02	2.06	2.05	2.03	2.03
BrBDBT	2.01	2.03	2.02	2.06	2.05	2.03	2.04
XA	2.00	2.04	2.02	2.03	2.03	2.03	2.04
AR	2.00	2.03	2.01	2.02	2.02	2.02	2.03
AD	2.00	2.01	2.01	2.02	2.02	2.02	2.03
AVE[(a)]	2.01	2.03	2.02	2.04	2.04	2.03	2.04

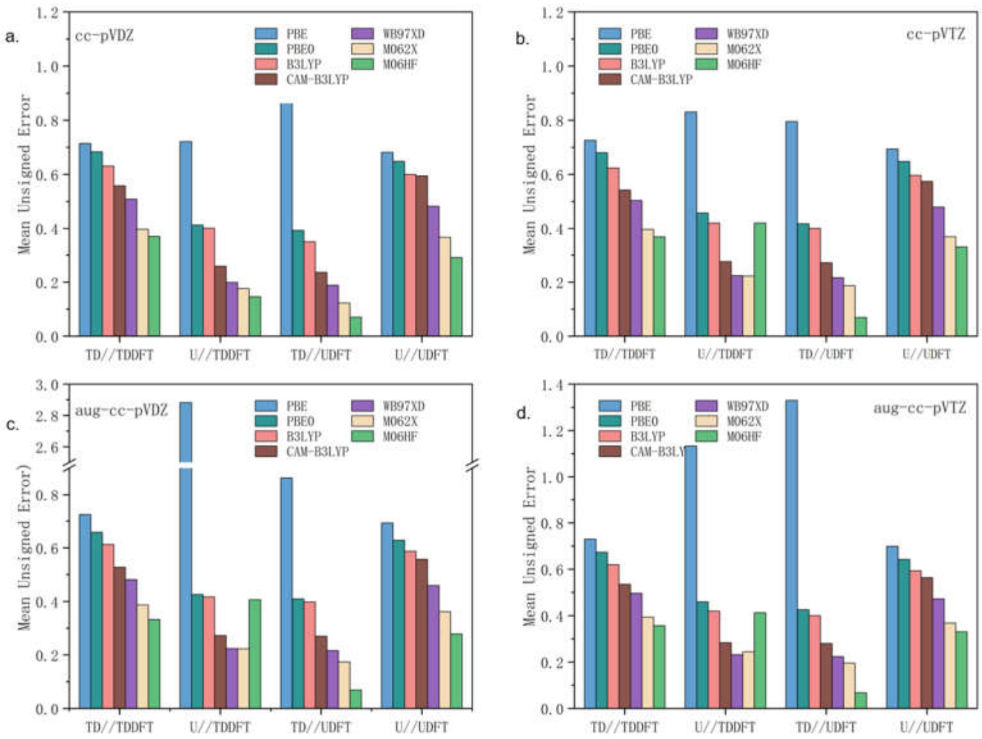

Figure S1: The MUE values (excluding BDBT, FBDBT, ClBDBT, BrBDBT) obtained by different basis sets under the same phosphorescence energy calculation method and functionals. (a), (b), (c), and (d) represent the results obtained with the basis set of cc-pVDZ, cc-pVTZ, aug-cc-pVDZ, and aug-cc-pVTZ, respectively.
